# Optimized Lipid Nanoparticles with Tail‐Modified Ionizable Lipids for Safer mRNA Delivery

**DOI:** 10.1002/advs.76539

**Published:** 2026-07-14

**Authors:** Seo‐Hyeon Bae, Jisun Lee, Jae‐Hun Ahn, Hosam Choi, Huijeong Choi, Jungmin Kim, Minho Kang, Ho Rim Oh, So Hyun Park, Eun Young Oh, Sanghyuk Jeon, Yeeun Lee, Yu‐Sun Lee, Na‐Young Lee, Hee‐Jin Bae, Jina Kwak, Jooil Kim, Nakyung Lee, Beomsun Kang, Ji‐Hye Kim, Eunsaem Song, Daesub Song, Hyunho Yoon, Sang‐Myeong Lee, Hyewon Youn, Kiyoun Lee, Byeong‐Cheol Kang, Jae‐Hwan Nam

**Affiliations:** ^1^ Department of Medical and Biological Sciences The Catholic University of Korea Bucheon Gyeonggi‐do Republic of Korea; ^2^ BK21 Plus Department of Biotechnology The Catholic University of Korea Bucheon Republic of Korea; ^3^ Department of Experimental Animal Research Biomedical Research Institute Seoul National University Hospital Seoul Republic of Korea; ^4^ Department of Chemistry The Catholic University of Korea Bucheon Republic of Korea; ^5^ Department of Biomedical‐Chemical Engineering The Catholic University of Korea Bucheon Gyeonggi‐do Republic of Korea; ^6^ Research Institute for Controlled Biomaterials of Regulated Cell Death The Catholic University of Korea Bucheon Republic of Korea; ^7^ Department of Nuclear Medicine Cancer Imaging Center Seoul National University Hospital Seoul Republic of Korea; ^8^ Cancer Research Institute Seoul National University College of Medicine Seoul Republic of Korea; ^9^ College of Veterinary Medicine Chungbuk National University Cheongju‐si Chungcheongbuk‐do Republic of Korea; ^10^ Department of Veterinary Pathology and Research Institute of Veterinary Science College of Veterinary Medicine Seoul National University Seoul Republic of Korea; ^11^ Graduate School of Translational Medicine Seoul National University College of Medicine Seoul Republic of Korea; ^12^ Department of Virology College of Veterinary Medicine and Research Institute For Veterinary Science Seoul National University Seoul Republic of Korea; ^13^ National Institute of Health Korea Disease Control and Prevention Agency Cheongju‐si Chungcheongbuk‐do Republic of Korea

**Keywords:** Ionizable lipid, Lipid nanoparticles, mRNA vaccine, Nonhuman primate model, Safety

## Abstract

Despite the growing importance of lipid nanoparticles (LNPs) in mRNA therapeutics, current ionizable lipids, which comprise an ionizable head group, a linker, and hydrophobic tails that collectively govern the delivery behavior, exhibit dose‐limiting toxicity and suboptimal efficiency. Hydrophobic tail chemistry has been implicated in these limitations; however, a systematic structure–activity relationship (SAR) analysis is lacking. In this study, the authors synthesized a 56‐member library of tail‐modified ionizable lipids with biodegradable disulfide linkages and variations in chain length and branching. Systematic SAR evaluation revealed that hydrophobic tail architecture critically determines endosomal escape, biocompatibility, and mRNA translation efficiency. Optimized LNPs delivered mRNA with potent efficacy and markedly reduced cytotoxicity, lower cytokine induction, and balanced Th1/Th2 immune activation in a nonhuman primate model. The findings establish the first comprehensive SAR framework linking tail chemistry to ionizable lipid function, offering molecular design principles for next‐generation LNPs and enabling the development of safer and more effective mRNA‐based vaccines and therapeutics.

## Graphical abstract

Systematic engineering of hydrophobic tail architecture in vitamin B5‐derived ionizable lipids establishes a comprehensive structure–activity relationship framework for mRNA delivery. Combined lipidtail and formulation optimization identifies TM1‐OPT3‐C, a lipid nanoparticle platform that improves efficacy–safety balance through efficient mRNA delivery, controlled immune activation, and reduced toxicity in nonhuman primates.

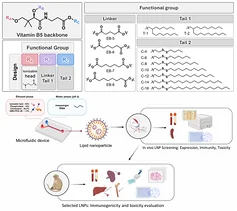

## 1 Introduction

Lipid nanoparticles (LNPs) have become a standard platform for delivering nucleic acids and therapeutic agents, establishing RNA‐based medicines as foundational components of contemporary drug development. The adoption of mRNA vaccines reinforces the essential role of LNPs in modern vaccinology and therapeutic applications [1, 2, 3]. LNPs provide an efficient platform for delivering genetic material, small molecules, and proteins, with applications in prophylactic vaccines, gene therapy, cancer immunotherapy, and treatments for genetic and infectious diseases [3, 4]. In this context, not only efficient intracellular delivery but also controlled immunogenicity and biocompatibility are critical determinants of therapeutic and vaccine efficacy. Composed of ionizable lipids, phospholipids, cholesterol, and PEGylated lipids, LNPs exhibit high encapsulation efficiency (EE), structural stability, and extended circulation time [3].

Central to LNP functionality are ionizable lipids, which remain neutral at physiological pH to reduce toxicity but acquire a positive charge in acidic endosomal environments. This pH‐responsive behavior facilitates complexation with negatively charged nucleic acids, promotes endosomal escape, and ensures efficient cytoplasmic  release of nucleic acids [5]. Despite progress, optimizing intracellular delivery efficiency while minimizing toxicity remains critical in mRNA therapeutic development [3]. Traditional cationic lipids exhibited high toxicity and poor biocompatibility, driving the design of ionizable lipids with improved safety and delivery performance [3, 5].

We explored ionizable lipids derived from vitamin B5, a water‐soluble vitamin and precursor of coenzyme A, known for its low toxicity and cytoprotective roles against oxidative and mitochondrial stress [6, 7, 8]. We developed the first vitamin B5‐based ionizable lipid (I82), designed as a safer alternative, but it showed unexpected toxicity and limited mRNA delivery efficiency [9, 10]. This outcome underscored the need for structural refinement, particularly of hydrophobic tail domains, which are now recognized as key determinants of ionizable lipid biocompatibility and delivery performance [9, 10]. Thus, the vitamin B_5_ scaffold alone was insufficient to ensure both efficient mRNA delivery and favorable tolerability, prompting us to focus on hydrophobic tail engineering as a rational strategy to optimize this lipid series.

The structure–activity relationships (SAR) of ionizable lipids, especially tail modifications, are key to advancing LNP design. Variations in hydrophobic tail chemistry, including saturation, unsaturation, and branching, have been shown to substantially affect delivery efficiency and immunogenicity [3, 11, 12]. Despite its recognized importance, studies on systematic SAR focusing on hydrophobic tail architecture in vitamin‐derived ionizable lipids remain limited. This knowledge gap has hindered the rational optimization of vitamin‐based ionizable lipids for mRNA delivery applications. In line with previous studies across multiple ionizable lipid scaffolds, tail‐related features such as branching, biodegradable motifs, and hydrophobic chain optimization are now recognized as important determinants of delivery efficiency, tolerability, and biodistribution [13, 14, 15]. Consistent with these reports, our study extends this design perspective to a vitamin B5‐derived scaffold and systematically examines how tail architecture contributes to biological performance within this lipid series.

In this study, we aimed to (i) systematically engineer the hydrophobic tail architectures of vitamin B5‐based ionizable lipids, (ii) elucidate their SAR, and (iii) identify optimized LNP formulations with improved mRNA delivery efficiency and safety. To achieve this, we modified tail structures and optimized overall LNP compositions, including helper lipids, cholesterol, and PEGylated lipids, to fine‐tune physicochemical properties and biological performance [11, 16]. These modifications enabled the identification of top‐performing LNPs exhibiting enhanced particle size, zeta potential, EE, and biocompatibility. In vitro and in vivo evaluations demonstrated superior mRNA translation, favorable biodistribution, and balanced immune responses, with reduced toxicity compared to conventional formulations [12, 17, 18].

Beyond identifying an optimized formulation within this series, our work provides scaffold‐specific design insights into how hydrophobic tail architecture influences ionizable lipid performance in a vitamin B5‐derived system. Through SAR analysis and complementary compositional optimization, we determined how tail architectures and lipid component ratios modulate endosomal escape, mRNA translation, biodistribution, and immune activation [16, 19]. This strategy addressed the limitations of earlier vitamin B5‐based lipid, yielding LNPs with improved delivery efficiency, safety, and immunogenicity [10]. These findings demonstrate that rational tuning of hydrophobic tail chemistry enables predictable modulation of LNP potency, biocompatibility, and tissue selectivity, while emphasizing the importance of SAR‐driven LNP design [11, 19]. Furthermore, our study provides insights into how tail architecture governs ionizable lipid behavior and establishes a framework for developing next‐generation mRNA therapeutics.

## 2 Results and Discussion

### 2.1 Design and Evaluation of Tail‐Modified Disulfide Ionizable Lipids for Enhanced mRNA Expression

The need for more efficient and safer LNPs for mRNA delivery motivated the design of novel tail‐modified (TM) ionizable lipids based on a vitamin B_5_ (D‐pantothenic acid) scaffold, a naturally occurring compound with functional groups amenable to chemical modification [8, 9]. This scaffold was selected as the backbone for constructing the TM ionizable lipids. The dimethylamino group was chosen as the tertiary amine ionizable head due to its demonstrated effectiveness in LNP formulation and delivery, and was conjugated to the primary alcohol of the B_5_ backbone via ester linkages, creating a platform for systematic tail variation [11, 20, 21].

Enhancement of biocompatibility while maintaining delivery efficiency was achieved by incorporating biodegradable disulfide bonds into the hydrophobic tails [17]. In this context, our findings highlight that scaffold‐specific tail optimization within a vitamin B5‐derived lipid system can improve biocompatibility while maintaining effective in vivo performance, supporting the value of this platform as a balanced efficacy‐safety‐oriented mRNA‐LNP system rather than a formulation designed solely to maximize immune output. These linkages enable reductive cleavage within cells, facilitating the release of mRNA payloads and minimizing lipid accumulation [17]. Studies have highlighted the importance of branched tails and conical tail geometries in improving LNP delivery efficiency [12, 22, 23]. The conical tail configuration was preserved, with one symmetric branched alkyl chain substituted to balance safety and efficacy. Tail modifications were introduced at Rʙ and R_C_. At Rʙ, four EB variants (EB‐5 to EB‐8) and two Ts variants (T‐1 and T‐2) were introduced, while at R_C_, seven disulfide‐containing tails with varying alkyl chain lengths, designated C2(S–S)C2*X* (*X* = 2–8), were introduced (Figure 1a, left panel). This approach yielded a library of 56 distinct ionizable lipids.

**FIGURE 1:**
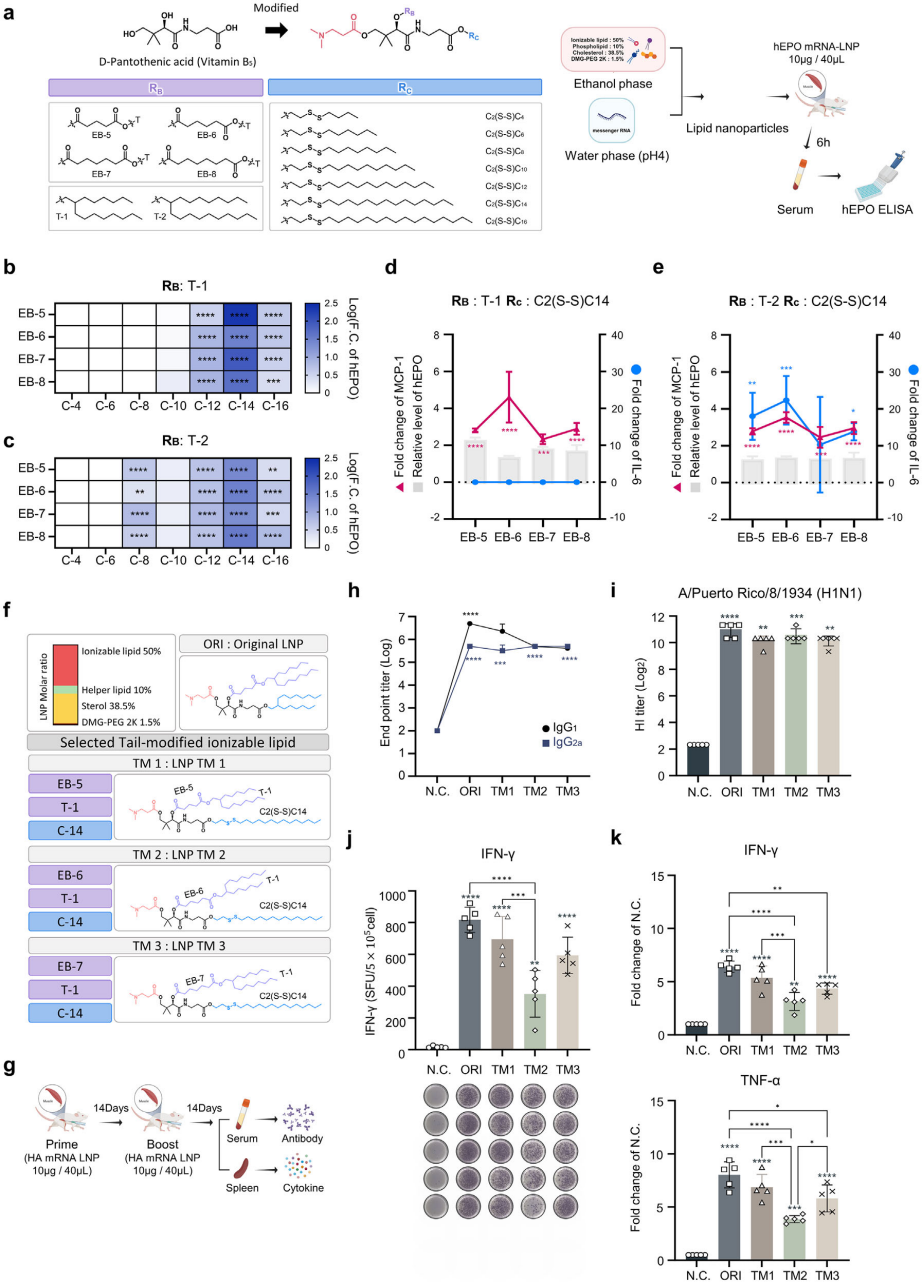
Formulation of lipid nanoparticles (LNPs) using 56 tail‐modified (TM) ionizable lipids and in vivo analyses of mRNA expression and immune responses of selected ionizable lipids. (a) Schematic illustration of TM ionizable lipids, the hEPO mRNA‐LNP formulation using these ionizable lipids, and the corresponding immunization schedule for hEPO/LNPs. Seven‐week‐old ICR mice were immunized with 10 µg/40 µL of hEPO/LNPs, and blood was collected 6 h post‐immunization for serum isolation and enzyme‐linked immunosorbent assay (ELISA). (b–e) For all experiments, results were compared by calculating the fold change relative to the values of the negative control (NC) group included in each experiment. (b, c) Relative expression levels of hEPO. (d, e) Relative levels of serum MCP‐1 and IL‐6. (f) Brief schematic diagram showing the three ionizable lipids and the molar ratio of the selected LNPs for systematic analysis. (g) Overview of the immunization schedules. Mice were immunized with saline or HA mRNA‐LNPs (10 µg/40 µL) as a prime dose, followed by a booster of the same dose 2 weeks later to compare immune responses. Serum samples were collected 2 weeks after the booster. (h) Serum IgG1 and IgG2a levels measured using ELISA. (i) Hemagglutination inhibition (HI) titers against vaccine strains measured in serum using the HI assay. (j) Numbers of HA peptide‐specific IFN‐γ‐secreting cells in splenocytes measured using ELISpot. (k) IFN‐γ and TNF‐α concentrations measured in splenocyte culture supernatants using ELISA. Data are reported as the mean ± standard deviation (SD). Statistical significance was analyzed using one‐way ANOVA. Differences were considered significant at *p < 0.05, **p < 0.01, ***p < 0.001, and ****p < 0.0001. Asterisks without connecting lines indicate comparisons with the NC group. All graphical illustrations were created using BioRender.com. (ORI: Original LNP).

LNP formulations were prepared using these lipids based on a standard mRNA delivery composition of an ionizable lipid, 1,2‐distearoyl‐sn‐glycero‐3‐phosphocholine (DSPC), cholesterol, and PEG‐lipid (DMG‐PEG 2K) at molar ratios of 50:10:38.5:1.5, respectively [3, 24]. LNPs were produced via microfluidic mixing by combining an ethanol‐phase lipid solution with an aqueous‐phase human erythropoietin (hEPO) mRNA solution at an optimized pH of 4.0, with each formulation containing 10 µg hEPO mRNA in 40 µL [25, 26]. After intramuscular administration, serum samples were collected at 6 h, and an enzyme‐linked immunosorbent assay (ELISA) was performed to quantify protein expression levels (Figure 1a, right panel).

Expression profiles varied according to the type and length of the hydrophobic tails (Figure 1b,c). For the Rʙ tail, expression levels varied by T type, regardless of the R_C_ type. When the C2(S–S)C‐2*X* (*X* = 2–8) tail at R_C_ did not exceed a certain length, formulation and expression efficiency were compromised. T‐1, with shorter carbon chains, required a longer hydrophobic tail for adequate formulation. Log fold change analysis for T‐1 revealed that combinations of EB‐5 and EB‐8 with shorter R_C_ tails (C‐4 to C‐8) were nonreactive. At R_C_ = C‐10, EB‐5, EB‐6, and EB‐7 demonstrated minimal expression, while EB‐8 showed slightly increased expression. When paired with R_C_ = C‐12, EB‐5 showed low expression, whereas EB‐6 to EB‐8 yielded moderate expression. The highest expression occurred with EB‐5 and EB‐7 combined with R_C_ = C‐14, followed by EB‐6 + C‐14 and EB‐8 + C‐14, exceeding the levels of EB‐6 to EB‐8 + C‐12. At R_C_ = C‐16, EB‐5, EB‐6, and EB‐7 showed moderate expression, similar across these EB types but lower than that of EB‐6 to EB‐8 + C‐12. EB‐8 paired with R_C_ = C‐16 exhibited expression comparable to EB‐5 + C‐12. T‐1 and T‐2 showed peak expression with C2(S–S)C‐14, followed by a decline with C2(S–S)C‐16 (Figure 1b,c). This pattern reflects the balance between hydrophobicity and ionizable lipid integration into the LNP lipid core. These findings confirm that optimal hydrophobic tail length is critical for LNP performance, consistent with previous studies [18].

Analysis of Rʙ tail variants in LNPs with the optimal C2(S–S)C‐14 tail revealed obvious differences between T‐1 and T‐2 types. In T‐1, hEPO expression varied among EB variants, with EB‐5 exhibiting the highest, EB‐6 the lowest, and EB‐7 and EB‐8 showing intermediate expression. MCP‐1 levels, indicating innate immune activation, peaked with EB‐6, followed by EB‐5 and EB‐8, and were lowest with EB‐7. MCP‐1 recruits monocytes and immune cells to sites of inflammation [27, 28, 29]. IL‐6 concentrations, which mediate inflammatory responses, remained low across all EB variants, suggesting limited pro‐inflammatory toxicity in T‐1 [30] (Figure 1d).

A comparison of EB‐5 through EB‐8 revealed that, despite similar expression and immunogenicity profiles, EB‐8 possesses a longer eight‐carbon Rʙ tail, conferring greater hydrophobicity than the shorter tails in EB‐5 to EB‐7. Excessive hydrophobicity disrupts lipid packing, reduces particle stability, alters biodistribution, and impairs therapeutic delivery, especially when no additional benefit in expression or immune modulation is observed [18, 19, 22]. Therefore, EB‐8 was excluded from further evaluation, while EB‐5, EB‐6, and EB‐7 were retained. EB‐5 exhibited relatively high hEPO expression with moderate immune activation; EB‐6 delivered high inflammatory signaling with reduced expression; and EB‐7 presented an intermediate phenotype (Figure 1b,d).

In the T‐2 group, hEPO expression and MCP‐1 levels were uniform across EB variants, indicating consistent delivery efficiency and moderate innate immune activation. However, elevated IL‐6 levels observed across all EB variants suggest a heightened pro‐inflammatory response that may pose immunotoxicity risks (Figure 1e). IL‐6 is a pro‐inflammatory cytokine that induces systemic inflammation, which can lead to adverse effects, including tissue damage and reduced tolerability of LNP formulations [30]. This immune activation underscores the need to balance expression potency with immune safety when designing ionizable lipid structures [29, 31].

To further investigate the physicochemical characteristics associated with differential mRNA delivery performance, the apparent pKa values of representative LNP formulations were measured using a 2‐(*p*‐toluidino)‐6‐naphthalene sulfonic acid (TNS) fluorescence assay (Figure S1). The measured apparent pKa values were 6.47 for SM‐102‐based LNP (SM), 5.73 for original LNP (ORI, Vitamin B_5_ scaffold‐based LNP), 5.39 for TM2, 5.22 for TM1, and 4.77 for TM3. These results demonstrate that both hydrophobic tail structure and LNP composition substantially influence the ionization behavior of the formulations under acidic conditions. Previous studies have reported that the apparent pKa of ionizable lipids is a critical determinant of endosomal protonation, membrane destabilization, and intracellular mRNA release efficiency [3, 32]. Interestingly, formulations exhibiting superior delivery performance and improved safety profiles, including TM1, showed intermediate acidic pKa values around 5.2, whereas TM3, which exhibited the lowest pKa value, showed relatively weaker functional performance. In contrast, SM and ORI exhibited comparatively higher pKa values, which may contribute to stronger protonation behavior but potentially increased nonspecific interactions and inflammatory responses. These findings suggest that balanced ionization properties within an optimal acidic pKa range may contribute to efficient mRNA delivery while maintaining favorable biocompatibility, further supporting the importance of rational SAR‐driven optimization of ionizable lipid structures and LNP composition.

eChemical variations among LNP components affect membrane properties such as lipid packing, fluidity, and stability, which influence immune activation and performance [24, 31]. We observed that subtle structural changes in ionizable lipids, particularly in hydrophobic tail length and conformation, alter mRNA expression and immune responses. EB‐5‐T1 achieved the optimal balance between delivery efficiency and immunogenicity, establishing the value of precise tail engineering. These findings highlight that fine‐tuning hydrophobic tail structure is essential for improving delivery performance while minimizing immune‐related toxicity, offering a clear strategy for next‐generation LNP design [11, 18]. Collectively, these results support an SAR within the vitamin B5‐derived lipid series, in which hydrophobic tail length, branching pattern, and disulfide‐containing tail architecture contribute to differences in formulation performance, mRNA expression, and inflammatory readouts. Notably, the highest‐performing structures were not defined simply by longer or more hydrophobic tails, but by a balanced tail architecture that supported efficient mRNA expression while limiting excessive cytokine induction. These observations are consistent with previous studies demonstrating that optimal mRNA delivery is often achieved through a balanced hydrophobic tail architecture rather than maximal hydrophobicity alone, suggesting that similar design principles may apply across multiple ionizable lipid scaffolds despite differences in head‐group chemistry [3, 33].

### 2.2 Characterization and Immune Response of Select Ionizable Lipids

Three TM ionizable lipids, TM1 (EB‐5‐T‐1‐C‐14), TM2 (EB‐6‐T‐1‐C‐14), and TM3 (EB‐7‐T‐1‐C‐14), were selected based on their promising mRNA expression and immune profiles (Figure 1f). Saline served as a negative control (NC) and the ORI served as a toxic positive control [9]. Since this study was designed to evaluate the effects of tail structure modification and subsequent formulation optimization within the vitamin B5 lipid scaffold, ORI was used as the primary reference formulation for comparisons within this lipid series. ORI exhibited an overall immunogenicity profile comparable to that of the SM (Figure S2).

Physicochemical characterization using dynamic light scattering revealed particle sizes of 100–150 nm, which are optimal for RNA vaccine delivery in mice and nonhuman primates [34] (Figure S3a). All formulations demonstrated low polydispersity indices (PDI < 0.2), indicating uniform and homogeneous nanoparticle populations [25]. Zeta potential measurements indicated near‐neutral surface charges (−12.5 to 12.5 mV) at physiological pH, a range generally stabilized by PEGylation and lipid composition [35, 36]. EE, assessed using fluorescent Quant‐it RiboGreen assay after Triton X disruption, showed values above 80% for all formulations, confirming effective mRNA loading comparable to that of SM [26] (Figure S3b).

Immunogenicity was assessed in BALB/c mice intramuscularly injected with hemagglutinin (HA) mRNA‐loaded LNPs. Vaccination consisted of a prime injection followed by a booster 14 days later, with serum and splenocyte collection performed 2 weeks after the booster (Figure 1g). Humoral immune responses were robust across HA mRNA‐treated groups, with significant increases in IgG1 and IgG2a antibody titers compared to saline controls (Figure 1h). IgG1 levels varied but not significantly, whereas IgG2a responses were consistent across the groups.

Neutralizing antibody titers, as measured by the hemagglutination inhibition (HI) assay, were significantly increased in all HA mRNA‐LNP‐treated groups compared with the negative control, with the highest titers observed in the ORI group (Figure 1i). Cell‐mediated immunity was evaluated using IFN‐γ enzyme‐linked immunospot (ELISpot) assays after HA peptide stimulation. All groups showed increases in IFN‐γ‐secreting splenocytes, confirming cellular activation (Figure 1j). However, TM2 elicited lower IFN‐γ responses compared to the ORI, and TM1, with a nonsignificant trend toward lower responses relative to TM3. TM1 and TM3 demonstrated IFN‐γ levels comparable to those of the ORI. Cytokine profiling of splenocyte supernatants showed elevated IFN‐γ and TNF‐α levels in all groups, with TM2 exhibiting reduced levels relative to other formulations (Figure 1k). These findings indicate that while all TM lipids effectively stimulate humoral immunity, tail branching affects cellular immune activation. TM2, despite higher neutralizing antibody titers, showed reduced Th1‐type cytokine responses, suggesting a bias toward humoral immunity. TM1 and TM3 exhibited a more balanced immune profile, highlighting the role of hydrophobic tail structure in tuning mRNA‐LNP vaccine immunogenicity.

### 2.3 Optimization of LNP Based on TM1 Ionizable Lipid (TM1‐Based LNP Formulations)

The physicochemical optimization of TM1‐based LNP formulations focused on varying the phospholipid‐to‐sterol lipid ratio while maintaining 45 mol% ionizable lipid content and 1.5 mol% DMG‐PEG 2K (Figure 2a, left panel) [37, 38]. Two phospholipids, DSPC and 1,2‐dioleoyl‐sn‐glycero‐3‐phosphoethanolamine (DOPE), were evaluated across five phospholipid‐to‐cholesterol ratios (OPT1 to OPT5), tested at N/P ratios of 3 and 6. hEPO mRNA‐loaded LNPs were prepared using a microfluidic device and administered intramuscularly at 10 µg per mouse. Serum was collected 6 h post‐injection for quantification of in vivo translation efficiency using hEPO ELISA (Figure 2a, upper right panel). Analysis of hEPO expression (Figure 2b) revealed higher translation efficiency at an N/P ratio of 6 than at 3 across formulations, consistent with findings that increased N/P enhances electrostatic stabilization of the mRNA‐ionizable lipid complex [37, 38]. Reduced sterol content enhanced expression, showing that excess sterol can increase membrane fluidity to levels that compromise LNP stability [22, 24]. Within DSPC‐based LNPs at N/P 6, expression was highest in OPT3, followed by near‐equivalent levels in OPT1 and OPT2, with OPT4 and OPT5 showing lower values. DOPE‐based LNPs at N/P 6 exhibited the highest expression in OPT2, followed by OPT1 and OPT3 at comparable levels, followed by OPT4 and OPT5. DOPE formulations outperformed DSPC counterparts, likely due to the cone‐shaped geometry of DOPE facilitating endosomal escape and mRNA release [39, 40]. At N/P 3, DSPC LNPs yielded measurable expression only in OPT1 and OPT2, while OPT3–5 produced near‐zero expression. In contrast, DOPE LNPs maintained a graded expression profile from OPT1 to OPT5 and outperformed DSPC formulations at the same ratio.

**FIGURE 2:**
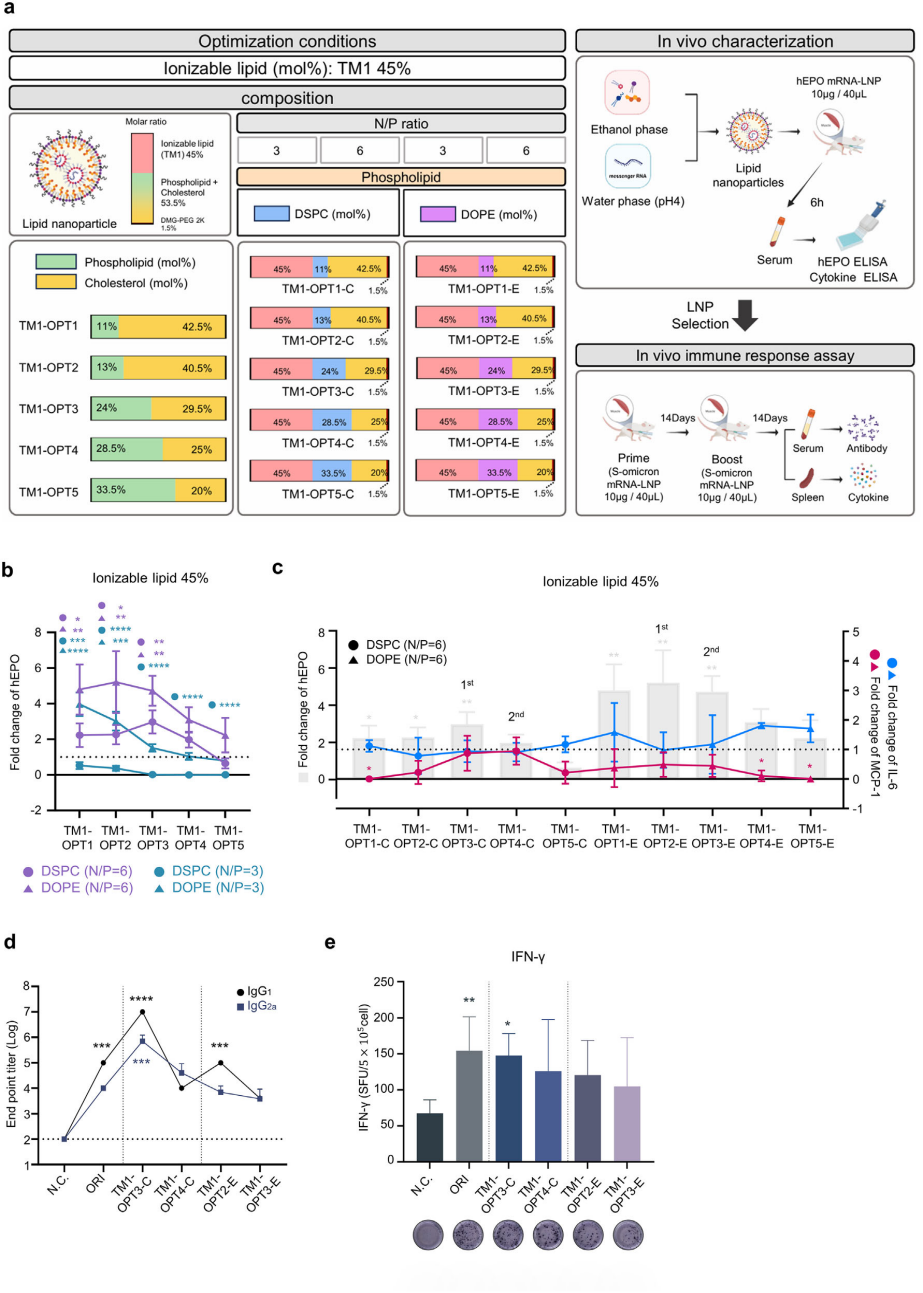
In vivo functional validation of selected rationally designed LNPs via mRNA expression, cytokine profiling, and vaccine‐induced immune responses. (a) Schematic illustration of the optimization conditions for the rational design of LNPs, the hEPO mRNA‐LNP formulation using ionizable lipids, and the corresponding immunization schedules for selected LNPs. Seven‐week‐old ICR mice were immunized with 10 µg/40 µL of hEPO mRNA‐LNPs, and blood was collected 6 h post‐immunization for serum analysis via ELISA. Mice were immunized with saline or S Omicron mRNA‐LNPs (10 µg/40 µL) as a prime dose, followed by a booster of the same dose 2 weeks later to compare immune responses. Serum samples were collected 2 weeks after the boost. (b, c) For all experiments, results were compared as fold changes relative to the values of the LNP ORI included in each experiment. (b) Relative expression levels of hEPO mRNA under different N/P ratios and LNP compositions. (c) Relative serum levels of MCP‐1 and IL‐6 across different LNP compositions at an N/P ratio of 6. (d) IgG1 and IgG2a levels measured in serum using ELISA. (e) Numbers of S Omicron peptide‐specific IFN‐γ‐secreting cells in splenocytes measured using ELISpot. Data are reported as the mean ± SD. Statistical significance was determined using one‐way ANOVA or two‐way ANOVA and considered significant at *p < 0.05, **p < 0.01, ***p < 0.001, and ****p < 0.0001. *p*‐value annotations indicate comparisons with the LNP ORI group in (b, c) and with the NC group in (d, e).

Early immune responses were evaluated by measuring serum MCP‐1 and IL‐6 levels (Figure 2c). In DSPC formulations, MCP‐1 levels peaked with OPT3‐C, followed by OPT4‐C, whereas IL‐6 levels remained low across all conditions, reflecting the role of DSPC in forming tight bilayers that reduce cytokine induction [31, 40]. In DOPE‐based LNPs, MCP‐1 was the highest in OPT2‐E, followed by OPT3‐E, whereas IL‐6 remained low but increased as sterol content decreased, consistent with the fusogenic effect of DOPE enhancing immune activation; cholesterol counteracted this effect by stabilizing the membrane [22, 39]. Parallel experiments with formulations containing 25% TM1 ionizable lipid, prepared across five different phospholipid‐to‐cholesterol ratios for DSPC‐ and DOPE‐based LNPs, yielded no suitable candidates. These formulations exhibited low MCP‐1 levels, indicating weak immune stimulation, with variable IL‐6 levels, reflecting inconsistent inflammatory responses (Figure S4). This supports prioritizing formulations with 45% ionizable lipid content within the OPT1–OPT5 design space [37, 38].

Phospholipid type, sterol ratio, and N/P ratio affect mRNA expression efficiency and innate immune activation. DOPE‐based LNPs produced higher protein expression than DSPC formulations, particularly at an N/P ratio of 6. MCP‐1 and IL‐6 cytokine profiles reflected the effects of lipid composition on immune responses [22, 31]. Based on these findings, TM1‐OPT3‐C, TM1‐OPT4‐C, TM1‐OPT2‐E, and TM1‐OPT3‐E formulations were selected for subsequent analyses because of their balanced expression and immune activation. These results establish a foundation for the evaluation of TM1‐based LNPs in downstream immunogenicity and efficacy studies. Collectively, these findings indicate that TM1‐based LNP performance was influenced not only by hydrophobic tail structure but also by formulation‐dependent physicochemical parameters, including particle size, polydispersity, zeta potential, EE, phospholipid identity, sterol content, and N/P ratio. Accordingly, the differences in delivery efficiency and mRNA expression among TM1‐based formulations should be interpreted as the combined outcome of ionizable lipid structure and overall LNP composition.

### 2.4 In Vivo Evaluation of Immunogenicity and Acute Toxicity of Selected TM1‐Based LNP Formulations

Based on optimization studies of phospholipid‐to‐sterol ratios, ionizable lipid content, and N/P ratios, four TM1‐based LNP formulations, TM1‐OPT3‐C, TM1‐OPT4‐C, TM1‐OPT2‐E, and TM1‐OPT3‐E, were selected for in vivo immunogenicity assessment due to their balance of mRNA expression and innate immune activation (Figure 2c, marked as first and second place, respectively). Physicochemical characterization of these formulations, with the SM, ORI, and TM1, assessed particle size (*z*‐average), PDI, zeta potential, and EE (Figure S5). ORI, TM1, and TM1‐OPT4‐C exhibited significantly larger particle sizes compared to the SM, whereas all formulations demonstrated narrow size distributions, with PDI values below 0.2, indicating homogeneous populations [24]. Zeta potential analysis showed that only TM1‐OPT3‐C and TM1‐OPT4‐C had significantly negative surface charges relative to zero, potentially affecting stability and cellular uptake [24, 35, 36]. EE was high across most formulations, exceeding 80%, except for TM1‐OPT3‐E, which showed an EE below this threshold, potentially compromising delivery efficacy [26, 34]. These characteristics contribute to differences in immunogenicity and underscore the importance of formulation characterization [31, 34].

To evaluate adaptive immune responses, mRNA encoding the spike protein of the SARS‐CoV‐2 Omicron variant (S Omicron) was encapsulated at 20 µg per dose into these formulations and administered intramuscularly to 6‐week‐old mice. N.C groups received Dulbecco's phosphate‐buffered saline (DPBS), and the ORI group received original Vitamin B_5_ scaffold‐based LNP. Mice were primed and boosted 14 days apart, with serum and splenocytes harvested 14 days post‐boost for comprehensive immunological analyses (Figure 2a, lower right panel).

Endpoint antibody titration assays revealed that most LNP formulations elicited robust IgG responses, significantly exceeding those of the N.C group (Figure 2d). Despite lower in vivo expression levels, DSPC‐based LNPs induced higher IgG titers than their DOPE‐based counterparts. The structural stability conferred by the tight bilayer architecture of DSPC improves immunogenicity by promoting effective antigen presentation and sustained immune stimulation [31, 40]. This aligns with findings that DSPC improves mRNA accumulation in lymphoid tissues such as the spleen, a key organ for initiating adaptive immunity [19]. In addition, the ability of DSPC to promote cytokines such as IL‐12 likely supports Th1‐type responses critical for protective immunity [41]. In contrast, the greater membrane fusion capacity of DOPE appears insufficient to offset its weaker structural integrity, limiting delivery to immune‐inductive sites [31, 39].

IgG subclass response analysis supported these observations (Figure 2d). ORI increased IgG1 levels without significantly altering IgG2a levels. TM1‐OPT3‐C significantly elevated both IgG1 and IgG2a, indicating a balanced Th1/Th2 response. TM1‐OPT4‐C elicited an increase in IgG1, whereas TM1‐OPT2‐E elicited a significant but lower IgG1 increase than TM1‐OPT3‐C. TM1‐OPT3‐E elicited an IgG1 response comparable to or lower than TM1‐OPT4‐C. TM1‐OPT4‐C, TM1‐OPT2‐E, and TM1‐OPT3‐E exhibited reduced IgG2a titers, highlighting variability in Th1‐skewed immunity among the formulations. Neutralizing antibody titers exhibited significant increases in ORI and the four TM1‐LNP formulations relative to the NC, with TM1‐OPT4‐C exhibiting slightly lower antibody titers (Figure S6). This synergy between cholesterol and DSPC maintains LNP integrity and promotes mRNA stability, optimizing delivery for enhanced stability and immune stimulation [19, 42].

T‐cell immunity, assessed using IFN‐γ ELISpot, was significantly elevated in ORI and TM1‐OPT3‐C‐treated groups compared with controls, whereas TM1‐OPT4‐C, TM1‐OPT2‐E, and TM1‐OPT3‐E showed increased but statistically insignificant responses (Figure 2e). These results indicate that cholesterol is critical for eliciting cellular immunity, with DSPC properties enhancing T‐cell activation [19, 42, 43]. Robust T‐cell responses are essential for protection and viral clearance, emphasizing the value of formulations that modulate adaptive immunity.

TM1‐OPT3‐C emerged as the lead formulation, demonstrating effective humoral and cellular immune responses while showing a favorable tolerability profile within the TM1‐based formulation series. These findings highlight the importance of optimizing cholesterol and phospholipid combinations to balance mRNA delivery, immune activation, and safety‐related outcomes. They further suggest that factors beyond peak mRNA expression, including LNP stability and biodistribution, critically influence the overall vaccine performance of mRNA‐LNP systems. Intracellular trafficking behavior may represent another important determinant of mRNA delivery performance beyond the physicochemical properties evaluated in this study. To gain preliminary insight into formulation‐dependent trafficking dynamics, DiD‐labeled LNP formulations were visualized by confocal microscopy at multiple time points following cellular uptake (Figure S7). Interestingly, ORI and TM1 formulations exhibited prominent intracellular puncta formation between 6 and 12 h post‐treatment, whereas the optimized TM1‐OPT3‐C formulation showed delayed but sustained puncta accumulation predominantly between 12 and 16 h. These punctate structures are consistent with vesicular localization following endocytic uptake and suggest differences in intracellular trafficking kinetics among the formulations. Notably, despite differences in ionizable lipid tail structure, ORI and TM1 exhibited similar temporal trafficking patterns, whereas compositional optimization in TM1‐OPT3‐C altered intracellular accumulation kinetics.

Although these imaging studies do not directly quantify endosomal membrane disruption or cytosolic release, the delayed and sustained intracellular puncta accumulation observed in TM1‐OPT3‐C suggests altered intracellular trafficking behavior relative to ORI and TM1. Whether this distinct intracellular accumulation pattern is directly associated with enhanced endosomal escape remains to be further investigated. Previous studies have demonstrated that helper lipid composition, cholesterol content, and LNP topology can substantially influence intracellular trafficking behavior, endosomal escape efficiency, and nucleic acid delivery performance [44, 45]. Furthermore, DOPE‐containing formulations have been reported to facilitate membrane fusion and endosomal destabilization through non‐bilayer phase formation, whereas DSPC‐containing formulations generally confer greater structural stability [3]. Collectively, these findings suggest that, beyond ionizable lipid tail structure alone, optimization of helper lipid and sterol composition can substantially modulate intracellular trafficking dynamics associated with mRNA delivery performance.

In addition, stability assessment under different storage and stress conditions, including 4°C, room temperature, and 37°C, showed that the selected S Omicron mRNA‐loaded LNPs maintained comparable particle sizes, low PDI values, and EE over 14 days, supporting their colloidal and encapsulation stability (Figure S8).

Temporal stability and pH‐dependent structural behavior of the selected mRNA‐loaded LNP formulations were evaluated using cryogenic transmission electron microscopy (Cryo‐TEM) (Figures S9 and S10). All formulations maintained their structural integrity during storage at 4°C for up to 14 days, consistent with their stable particle size, PDI, and EE profiles. However, under acidic conditions of pH 5.0, distinct structural behaviors were observed among the formulations. While SM and ORI largely retained spherical morphologies comparable to those observed at physiological pH, TM1 and TM1‐OPT3‐C exhibited pronounced internal structural rearrangements characterized by displacement of the electron‐dense core and formation of bleb‐like surface protrusions. These pH‐dependent morphological transitions suggest protonation‐induced lipid reorganization under endosomal‐like conditions and may facilitate interactions with endosomal membranes, consistent with recent reports demonstrating that pH‐triggered structural remodeling of LNPs can promote membrane‐active conformations associated with intracellular nucleic acid delivery [46]. Collectively, these findings indicate that TM1‐based formulations possess both favorable colloidal stability and enhanced structural responsiveness to acidic environments, characteristics that may contribute to efficient intracellular mRNA delivery. Taken together with the physicochemical characterization, pKa analysis, and pH‐dependent structural remodeling studies presented herein, these results indicate that TM1‐based LNP performance is governed not only by hydrophobic tail architecture but also by multiple formulation‐dependent properties, including ionization behavior, colloidal stability, intracellular trafficking dynamics, and overall lipid composition. Further investigation will facilitate the rational design of next‐generation mRNA vaccines with a more balanced efficacy–safety profile [31, 40, 42, 43].

### 2.5 Acute Toxicity of Top‐Performing LNP Formulation in Mice

To compare the safety of the TM1 ionizable lipid with ORI, we conducted an acute toxicity study in mice. DPBS served as the NC, ORI as a toxic positive control, TM1 as a TM formulation, and TM1‐OPT3‐C as the optimized composition. Mice received two high‐dose intramuscular injections (50 µg mRNA‐LNP) at a 2‐week interval, and serum biochemical and hematological parameters were analyzed 2 days after the final dose (Figure 3a). Biochemical assays (Figure 3b–f) showed elevated aspartate transaminase (AST) and alanine transaminase (ALT) levels in ORI and TM1, indicating hepatocellular damage, whereas TM1‐OPT3‐C levels were comparable to those in the NC. High‐density lipoprotein cholesterol (HDL‐C) was significantly reduced only in ORI. Blood urea nitrogen (BUN) levels were significantly lower in all LNP‐treated groups than in the NC group. Among the LNP‐treated groups, ORI showed the lowest BUN level, whereas TM1‐OPT3‐C showed a slightly higher value than ORI and TM1, while remaining below that of the NC group. These results indicate that TM1‐OPT3‐C did not induce an apparent renal burden under the tested conditions. Glucose levels were lowest in ORI, reflecting metabolic impairment. Hematological data (Figure 3g–l) showed that red blood cell (RBC), hemoglobin, and hematocrit were highest in ORI, followed by TM1 and TM1‐OPT3‐C. Reticulocyte and lymphocyte counts were reduced in ORI and TM1 but preserved in TM1‐OPT3‐C. Platelet counts decreased across all groups without remarkable differences among formulations. TM1 and TM1‐OPT3‐C exhibited lower hepatic, renal, and hematopoietic toxicity than ORI, with TM1‐OPT3‐C showing the most favorable profile. The reduced toxicity in TM1 is attributed to the incorporation of a biodegradable disulfide bond in its tail structure, which enhances biocompatibility in reductive environments [17, 47]. The superior tolerability of TM1‐OPT3‐C highlights the importance of optimizing lipid composition to minimize LNP‐associated toxicity [19, 22, 31].

**FIGURE 3:**
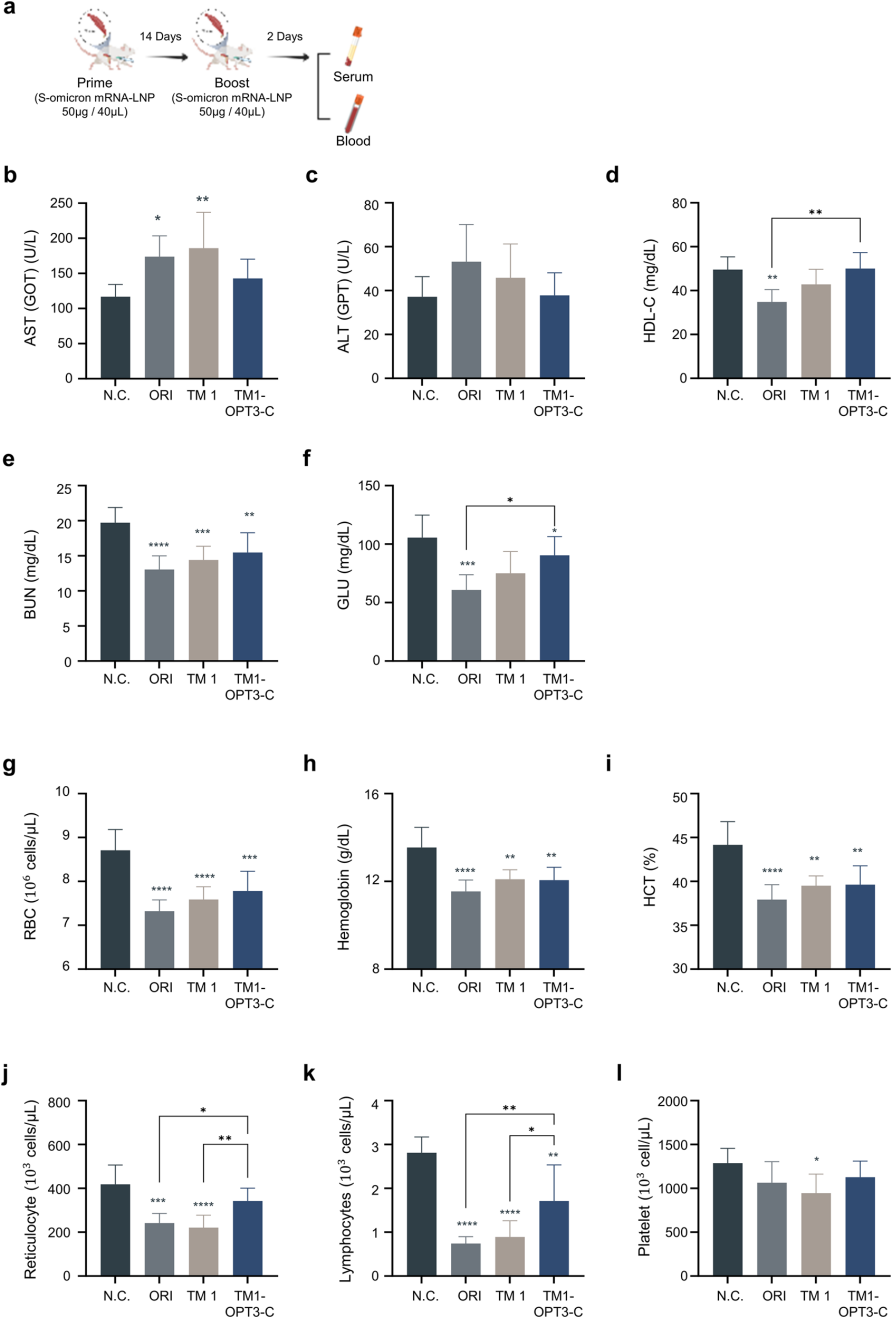
In vivo acute toxicity of top‐performing LNPs. (a) Overview of the immunization schedules. Mice were immunized with DPBS and S Omicron mRNA‐LNPs to evaluate toxicity. (b–f) Biochemical analysis of major toxicity indicators. (b) Alanine transaminase (ALT) levels. (c) Aspartate transaminase (AST) levels. (d) High‐density lipoprotein cholesterol (HDL‐C) levels. (e) Blood urea nitrogen (BUN) levels. (f) Glucose (GLU) levels. (g–l) Hematological analysis of major toxicity indicators. (g) Red blood cell (RBC) count. (h) Hemoglobin levels. (i) Hematocrit (HCT) percentage. (j) Reticulocyte count. (k) Lymphocyte count. (l) Platelet counts. Data are reported as the mean ± SD. Statistical significance was analyzed using one‐way ANOVA. Differences were considered significant at *p < 0.05, **p < 0.01, ***p < 0.001, and ****p < 0.0001.

### 2.6 Evaluation of mRNA‐LNP‐Induced Immunogenicity in Cynomolgus Macaques

We assessed the immunogenicity of the formulations in cynomolgus macaques using mRNA encoding SARS‐CoV‐2 spike protein (Figure 4a). Animals received four doses of the mRNA‐LNP formulations at 2‐week intervals, and blood, spleen, skeletal muscle at the injection site, liver, and sternal bone marrow samples were collected at predefined time points for immunological and histopathological assessments. Humoral immune responses were assessed by measuring spike‐specific total IgG titers at a serum dilution of 1:3200 (Figure 4b). TM1 and TM1‐OPT3‐C groups generated robust antibody titers comparable to those induced by ORI, suggesting that tail modification does not compromise B cell‐mediated immunity. Neutralizing activity against SARS‐CoV‐2 BA.4 and wild‐type strains was confirmed using neutralization assays (Figure 4c). TM1‐OPT3‐C demonstrated broader and more potent neutralizing capacity across serum dilutions, maintaining >50% neutralization activity at higher dilutions compared to ORI and TM1. This broad‐spectrum neutralization can be attributed to the formulation's improved antigen presentation efficiency and balanced immune profile observed in murine studies. Cellular immune responses were quantified using IFN‐γ ELISpot assays on splenocytes (Figure 4d). Upon peptide stimulation, the ORI and TM1‐OPT3‐C groups exhibited significantly higher IFN‐γ‐secreting T cell frequencies compared to the NC, whereas the TM1 group showed relatively lower activity. These results indicate that the TM formulation promotes antigen‐specific Th1‐biased cellular responses, consistent with previous reports linking optimized LNP compositions to enhanced T cell activation [31, 43]. Immunogenicity data from macaques corroborate our observations in murine models, showing that hydrophobic tail engineering in the TM1‐OPT3‐C configuration can enhance adaptive immunity while preserving safety. These findings align with evidence showing that alterations in lipid hydrophobicity and branching can modulate antigen‐specific T and B cell responses through effects on endosomal escape kinetics and mRNA translation efficiency [11, 12, 16, 18]. Given the translational relevance of the macaque model, these results support the clinical potential of TM1‐OPT3‐C as a next‐generation mRNA vaccine delivery platform.

**FIGURE 4:**
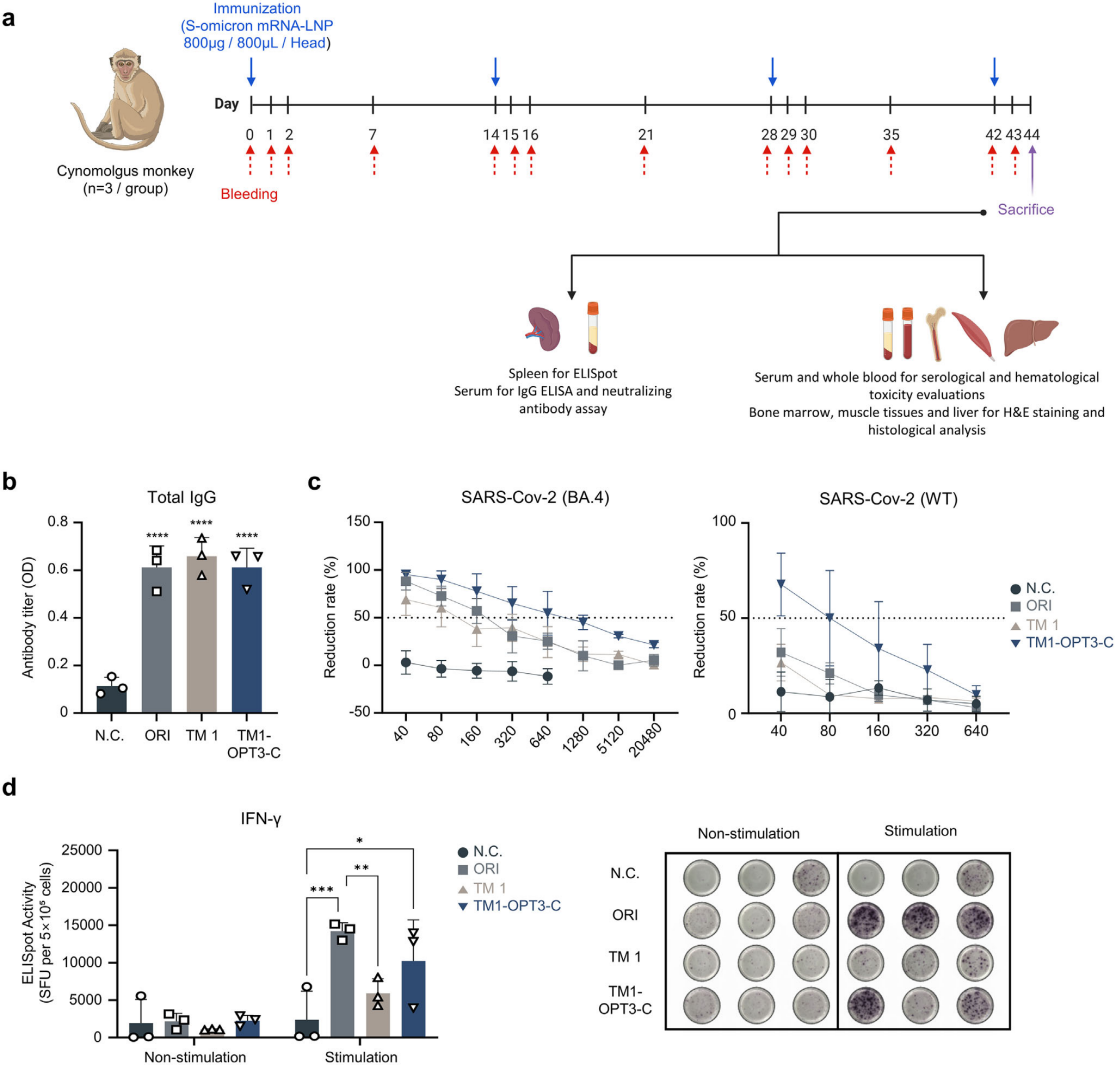
In vivo immune responses of selected top‐performing LNPs in nonhuman primates. (a) Overview of the immunization schedules. Female cynomolgus monkeys (*n* = 3 per group) were immunized four times at 2‐week intervals with saline or S Omicron mRNA‐LNPs. Two days after the final immunization, the animals were sacrificed, and spleen and serum were collected for immune analysis. (b) Total IgG levels measured using ELISA on plates coated with S Omicron protein. (c) Measurement of neutralizing antibody titers against SARS‐CoV‐2 (BA.4 and WT). (d) Numbers of S Omicron protein‐specific IFN‐γ‐secreting cells in splenocytes measured using ELISpot. Data are reported as the mean ± SD. Statistical significance was analyzed using one‐way ANOVA. Differences were considered significant at *p < 0.05, **p < 0.01, ***p < 0.001, and ****p < 0.0001.

### 2.7 Safety, Tolerability, and Tissue Distribution in Nonhuman Primates

To assess the safety of the optimized TM1‐OPT3‐C formulation compared to unoptimized TM1 and ORI, we conducted hematological, biochemical, and histopathological evaluations in cynomolgus macaques at the study endpoint (Day 44) (Figure 5). Serum biochemical marker analysis showed that C‐reactive protein (CRP) was significantly elevated in all groups compared to the NC (Figure 5a), indicating systemic inflammation associated with mRNA‐LNP administration [29, 31]. Lactate dehydrogenase (LDH) levels were highly increased in ORI (Figure 5b), whereas TM1 and TM1‐OPT3‐C showed lower LDH levels, indicating reduced cellular injury. Although ORI contains a vitamin B_5_ backbone, the tailored hydrophobic tail structures and optimized lipid composition in TM1 and TM1‐OPT3‐C reduced mitochondrial stress and ROS‐mediated injury, thereby improving biocompatibility compared to ORI [17, 47]. AST and ALT levels remained within normal ranges across all groups, with TM1‐OPT3‐C exhibiting the lowest ALT levels (Figure 5c,d), suggesting no severe hepatocellular injury. Liver weight was significantly higher in all groups compared to the NC (Figure 5e), possibly reflecting mild hepatomegaly from immune activation or metabolic stress [31].

**FIGURE 5:**
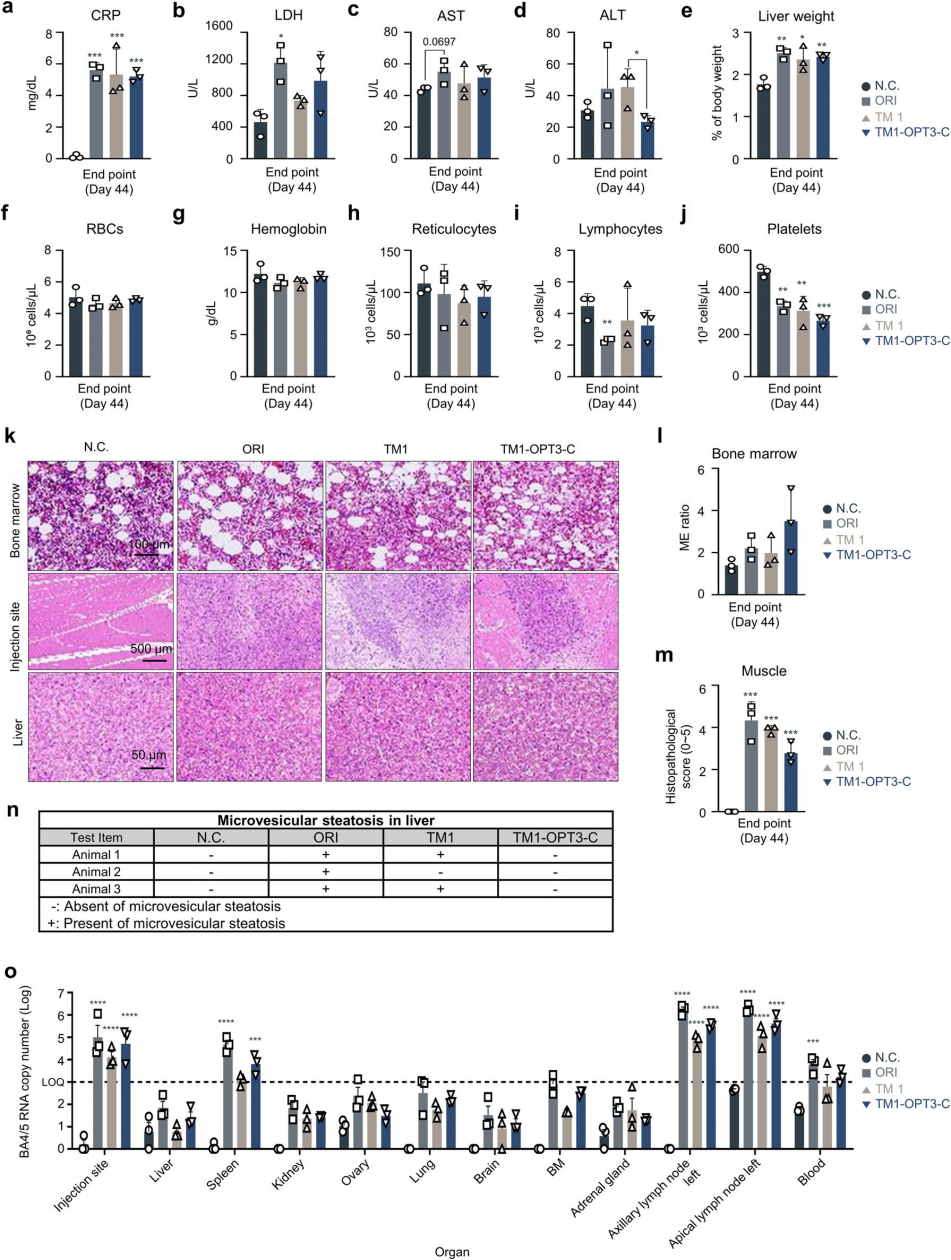
Safety and toxicological assessment of mRNA‐LNP formulations in cynomolgus macaques. (a–n) Female cynomolgus macaque were intramuscularly injected with DPBS or the indicated mRNA vaccine candidate (800 µg/head), four times at 2‐week intervals. The animals were euthanized 2 days after the final injection. (a–d) The indicated parameters were analyzed via blood chemistry. (e) Liver weight was normalized to body weight. (f–j) Levels of the indicated parameters were analyzed using complete blood count (CBC). (k) The indicated tissues were stained with hematoxylin and eosin (Bone marrow: sternum bone). (l) The myeloid to erythroid (M/E) ratio of the bone marrow tissue was manually calculated. (m) Histopathological severity at the injection site (brachialis muscle) was scored on a 0–5 scale (0: Not severe, 5: Most Severe). (n) Presence or absence of microvesicular steatosis in the liver of each animal is presented in the table. Data are reported as the mean ± SD (*n*  =  3), and statistically significant differences between the negative control and each test group were analyzed using one‐way ANOVA test followed by the Dunnett's post hoc test (*P < 0.05, **P < 0.01, **P < 0.001). (o) Biodistribution of mRNA across different organs. The limit of detection (LOD) was 10^2^, and the limit of quantification (LOQ) was 10^3^.

Hematological analysis demonstrated no significant differences in RBC counts, hemoglobin levels, or reticulocyte numbers among the groups (Figure 5f–h). However, lymphocyte and platelet counts were reduced in all groups compared to those in the NC (Figure 5i,j), indicating that the impact of mRNA‐LNP on hematopoiesis persisted with TM formulations. However, these effects reversed one week after immunization (data not shown).

Histopathological examination of bone marrow, injection site tissue, and liver revealed the local and systemic tolerability of mRNA‐LNP (Figure 5k). In the bone marrow, all inoculated groups showed increased cellular density compared to that in the NC, with TM1‐OPT3‐C showing the most pronounced effect, where reduced fat space and increased hematopoietic cellularity suggested hypercellularity or myeloid lineage expansion. However, no signs of toxicity, such as necrosis, fibrosis, or atypical cells, were observed. At the muscle injection site, all vaccinated groups showed inflammatory cell infiltration and tissue structural heterogeneity compared to the NC. However, this may be a normal immune response following vaccination.

The myeloid/erythroid ratio increased in all groups compared to that in the NC, with a modest elevation in TM1‐OPT3‐C, indicating innate immune activation after vaccination (Figure 5l). The muscle histopathological score (Figure 5m) increased in all vaccinated groups compared to that in the NC. However, TM1‐OPT3‐C showed a lower score than ORI, indicating less local muscle inflammation. Analysis of microvesicular steatosis in the liver (Figure 5n) showed that ORI had the highest hepatic lipid degeneration, while TM1‐OPT3‐C exhibited a safer hepatic profile. These histological findings in cynomolgus macaques indicate that TM1‐OPT3‐C exhibits lower hepatotoxicity than ORI, no systemic bone marrow toxicity, and expected injection site inflammation, suggesting improved biocompatibility and metabolic safety. The reduced inflammatory response and hepatic alterations observed with TM1‐OPT3‐C align with previous reports that optimized ionizable lipid structures, particularly through hydrophobic tail engineering, can reduce off‐target immune activation and improve tolerability without compromising efficacy [17, 31, 47].

To further evaluate the relationship between tolerability, mRNA expression, and carrier lipid fate, we performed parallel tissue biodistribution analyses of the antigen‐encoding mRNA and ionizable lipid component following intramuscular administration in cynomolgus macaques. Antigen mRNA (S Omicron BA.4/5) levels were quantified using quantitative reverse transcription polymerase chain reaction (RT‐qPCR). RT‐qPCR analysis revealed that across all LNP formulations, quantifiable mRNA signals were localized at the injection site and draining lymph nodes, particularly the left axillary and left apical lymph nodes (Figure 5o). This pattern reflects the expected pharmacological distribution of LNPs, where particles remain concentrated locally while a fraction traffics through the lymphatics into regional nodes, as reported in macaque studies examining LNP biodistribution following intramuscular injection [48, 49]. TM1‐OPT3‐C yielded signals above the limit of detection, confirming effective local and lymphatic exposure, although the levels were lower than those of ORI. In contrast, TM1 exhibited weaker signals in these tissues than the other LNPs. In the spleen, all three groups displayed low‐to‐moderate signals above the limit of quantification, reflecting uptake by splenic macrophages, consistent with previous reports that LNP surface chemistry influences spleen tropism via macrophage‐mediated uptake [50, 51].

In off‐target tissues, including the liver, kidney, ovary, lung, brain, bone marrow, adrenal gland, and blood, TM1 and TM1‐OPT3‐C yielded a higher proportion of non‐detectable (ND) samples, whereas ORI showed detectable signals across organs. These differences reveal that ionizable lipid architecture governs biodistribution and off‐target accumulation [51, 52, 53, 54]. ORI, with less optimized hydrophobic tails, formed less stable particles prone to systemic leakage, resulting in higher off‐target accumulation and toxicity. TM1, although refined, remained suboptimal, leading to poor lymphatic distribution. In contrast, TM1‐OPT3‐C combined tailored tail modifications with compositional optimization to achieve efficient lymphatic targeting and reduced systemic exposure, consistent with strategies such as SORT design that modulate tissue tropism via lipid composition [48, 50]. This selective distribution profile aligns with recent studies showing that optimized LNP formulations enhance tolerability and minimize off‐target exposure [49, 51].

These findings indicate that TM1‐OPT3‐C provides effective local and lymphatic delivery while minimizing off‐target distribution, maintaining safety in nonhuman primates, and demonstrating its potential as a safe next‐generation mRNA‐LNP platform with translational relevance.

### 2.8 TM1‐OPT3‐C Improves Local Safety and Reduces Mitochondrial and Inflammatory Stress

To elucidate the mechanistic basis for the enhanced tolerability of TM1‐OPT3‐C in nonhuman primates, we conducted a head‐to‐head comparison against an ALC‐0315‐based LNP (ALC) in mice (Figure 6a–c). Cryo‐TEM analysis revealed that both formulations displayed spherical morphology with a highly electron‐dense core. Both LNPs showed consistent lamellarity and comparable particle size and homogeneity, indicating no significant structural differences [24, 34] (Figure 6b). Following intramuscular administration of firefly luciferase mRNA encapsulated with LNPs, ex vivo bioluminescence imaging at 3 h revealed that TM1‐OPT3‐C elicited the highest splenic signal and lower off‐target expression in the liver, kidney, and lung. In contrast, the ALC showed reduced splenic expression but higher off‐target signals [19, 50] (Figure 6c,d). Although these findings reflect luciferase expression rather than direct lipid localization, they parallel the RT‐qPCR‐based antigen biodistribution observed in nonhuman primates, showing that TM1‐OPT3‐C achieves more targeted lymphoid expression with reduced systemic exposure [48].

**FIGURE 6:**
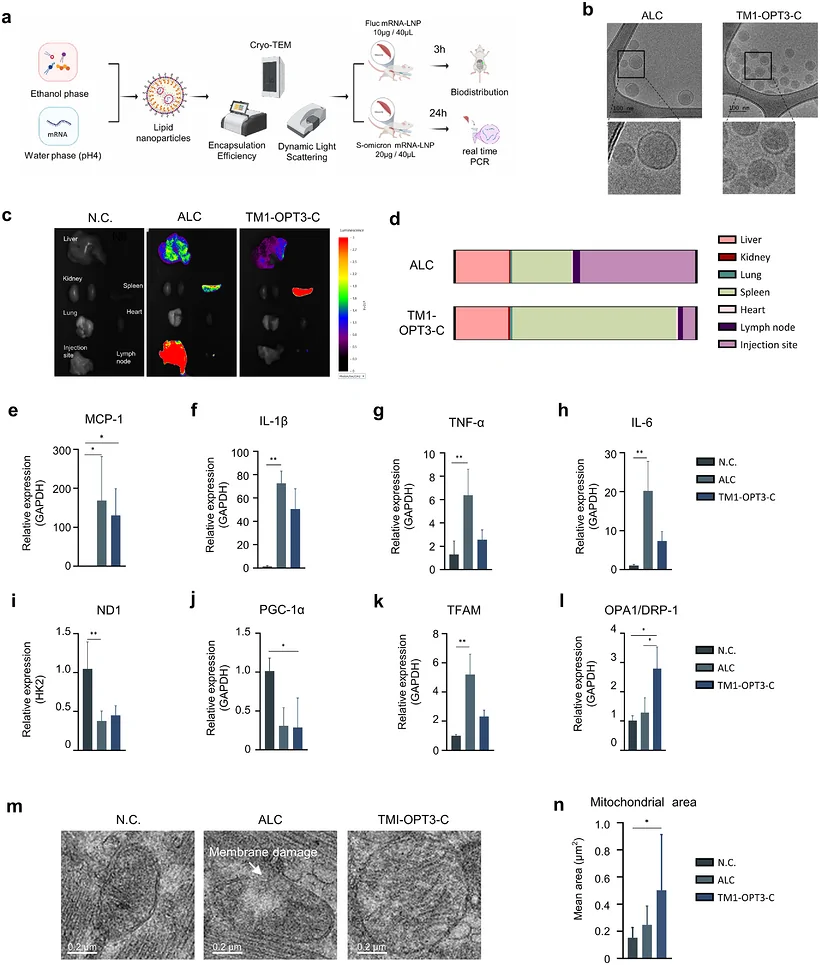
In vivo biodistribution and cellular stress assessment of mRNA‐LNP formulations in mice. (a) Schematic illustration of lipid nanoparticles, Fluc mRNA‐LNP, and S Omicron mRNA‐LNP, their physicochemical characterization and the corresponding immunization schedule for each formulation. Seven‐week‐old BALB/c female mice were immunized with DPBS and 10 µg/40 µL Fluc mRNA‐LNP, and organs (liver, kidney, lung, spleen, heart, lymph node, and injection site) were collected 3 h post‐immunization for biodistribution analysis. Seven‐week‐old C57BL6 female mice were immunized with DPBS and 20 µg/40 µL S Omicron mRNA‐LNPs, and the tissues from the injection site were collected 24 h post‐immunization for quantitative reverse transcription polymerase chain reaction (RT‐qPCR) analysis. (b) Cryo‐transmission electron microscopy (Cryo‐TEM) images of LNPs (scale bar: 100 nm). (c) Biodistribution images of Fluc mRNA‐LNPs. (d) Quantification of fluorescence intensity in major organs, expressed as relative distribution percentages. (e–l) Cellular stress response at the injection site following S Omicron mRNA‐LNP administration, assessed via RT‐qPCR. (e) Monocyte chemoattractant protein‐1 (MCP‐1). (f) Interleukin‐1 beta (IL‐1β). (g) Tumor necrosis factor‐alpha (TNF‐α). (h) Interleukin‐6 (IL‐6). (i) NADH dehydrogenase subunit 1 (ND1). (j) Peroxisome proliferator‐activated receptor gamma coactivator 1‐alpha (PGC‐1α). (k) Mitochondrial transcription factor A (TFAM). (l) OPA1/DRP‐1 ratio. (m) Representative Cryo‐TEM images for structural analysis of mitochondria (scale bar: 0.2 µm). (n) Mitochondrial area measured using cryo‐TEM. Data are reported as the mean ± SD. Statistical significance was analyzed using one‐way ANOVA. Differences were considered significant at *p < 0.05, **p < 0.01, ***p < 0.001, and ****p < 0.0001. (ALC: ALC‐0315‐based LNP).

Pro‐inflammatory cytokine expression highlighted differences in local immunoreactivity (Figure 6e–h). MCP‐1 (Figure 6e) and IL‐1β (Figure 6f) levels were comparable across groups. In contrast, TNF‐α (Figure 6g) and IL‐6 (Figure 6h) levels were elevated in the ALC group (*p* < 0.01), while TM1‐OPT3‐C maintained levels closer to baseline [29, 30].

Mitochondrial gene expression at the injection sites was examined to evaluate cellular stress responses (Figure 6i–k). ND1 (Figure 6i) and PGC‐1α (Figure 6j) showed modest decreases in the ALC and TM1‐OPT3‐C groups relative to the NC, suggesting that mRNA‐LNP administration may trigger basal mitochondrial stress [29, 55]. TFAM expression (Figure 6k) was significantly increased in the ALC group (*p* < 0.01), whereas TM1‐OPT3‐C did not induce such elevation, indicating that TM1‐OPT3‐C preserves physiological TFAM levels, thereby preventing excessive activation of mitochondrial transcriptional pathways observed with the ALC [56, 57]. Further evaluation of mitochondrial dynamics revealed differences among LNP formulations (Figure 6l–n). The OPA1/DRP1 ratio (Figure 6l), a molecular indicator of mitochondrial fusion–fission balance, remained unchanged in the ALC group but was significantly increased in TM1‐OPT3‐C, suggesting an adaptive mitochondrial response that may contribute to the stabilization of cristae architecture [58, 59]. TEM structural analysis (Figure 6m) showed that mitochondria from ALC‐treated muscle exhibited disrupted membranes and disorganized cristae, whereas TM1‐OPT3‐C exhibited intact double membranes and organized cristae structure, although the mitochondria appeared moderately enlarged. Quantitative analysis of mitochondrial area (Figure 6n) confirmed a significant increase in TM1‐OPT3‐C compared to the ALC, indicating adaptive remodeling and compensatory biogenesis rather than pathological swelling [60, 61]. These results suggest that TM1‐OPT3‐C promotes adaptive mitochondrial remodeling and maintains ultrastructural integrity, mitigating the fragmentation and oxidative stress patterns commonly induced by conventional ionizable lipids [55, 62].

These findings indicate that the ALC induces mitochondrial stress (ND1, TFAM) and inflammatory cytokine responses (TNF‐α, IL‐6), contributing to local mitochondrial dysfunction and tissue inflammation, whereas TM1‐OPT3‐C effectively suppresses these perturbations and promotes adaptive mitochondrial remodeling, reflecting superior biocompatibility [29, 55, 56, 63]. These findings align with our histopathological observations showing reduced immune cell infiltration at the injection site, further supporting the enhanced local safety profile of TM1‐OPT3‐C relative to the ALC.

The observed mitochondrial and cytokine responses are consistent with previous reports suggesting that LNP systems can perturb mitochondrial homeostasis and modulate innate immune signaling [29, 55]. For instance, in vitro studies have demonstrated the alleviation of LNP‐associated mitochondrial dysfunction through optimization of cargo or lipid composition [62, 64]. Moreover, multiple studies emphasize that ionizable lipid chemistry and overall LNP composition critically influence in vivo biodistribution, immune activation, and tolerability [51, 65]. Accordingly, strategies involving biodegradable or TM ionizable lipids have been proposed to minimize off‐target immune activation while enhancing biocompatibility [51, 65]. The observed increase in mitochondrial fusion markers, preserved ultrastructure, and reduced cytokine induction collectively indicate that TM1‐OPT3‐C stabilizes mitochondrial function and minimizes inflammatory stress, providing a mechanistic explanation for its improved tolerability compared with the benchmark ALC‐0315‐based LNP. Overall, these results support conclusion that fine‐tuning ionizable lipid tail chemistry and lipid ratios can mitigate mitochondrial perturbation and downstream inflammatory signaling, thereby improving local safety without compromising efficacy [11, 16, 18].

## 3 Conclusion

In this study, we demonstrated that optimization of LNPs through hydrophobic tail engineering and biodegradable disulfide linkages enhances the safety, tolerability, and tissue‐selective delivery of mRNA therapeutics. The optimized TM1‐OPT3‐C formulation achieved efficient mRNA delivery, minimized off‐target exposure, and reduced mitochondrial and inflammatory stress, collectively supporting a more balanced efficacy–safety profile compared with earlier or unoptimized LNPs. These findings highlight the advantage of scaffold‐specific tail optimization in improving biocompatibility while maintaining effective in vivo performance, thereby providing a foundation for next‐generation mRNA‐LNP therapeutics. Future studies should focus on scalability and broader disease applications that are critical for clinical translation.

## 4 Materials and Methods

### 4.1 Materials

SM‐102 was procured from Hanmi Fine Chemical Co., Ltd. (Siheung, Gyeonggi‐do, Republic of Korea). ALC‐0315, ALC‐0159, DOPE, DSPC, cholesterol, and 1,2‐dimyristoyl‐rac‐glycero‐3‐methoxypolyethylene glycol‐2000 (DMG‐PEG 2K) were obtained from Avanti Polar Lipids (Alabaster, AL, USA). The original vitamin B_5_‐core ionizable lipid was synthesized following established protocols [9]. TM ionizable lipids were synthesized in‐house at KL's Organic Synthesis Laboratory. Additional reagents for chemical synthesis were purchased from Sigma‐Aldrich (St. Louis, MO, USA) and Tokyo Chemical Industry (Tokyo, Japan) and used without further purification.

### 4.2 Synthesis of Ionizable Lipids

EB–T chain synthesis and a representative synthesis method for TM ionizable lipids are described in the Supporting Information (Figures S11–S19).

### 4.3 Preparation of mRNA

The DNA template for mRNA transcription contained 5′ and 3′ untranslated regions (UTRs), a multicloning site with four restriction enzyme sites, and a polyadenylation signal, as described previously [66]. This platform, designated CUK3‐1, was linearized with the NotI restriction enzyme, purified, and quantified spectrophotometrically. Genes of interest, including human erythropoietin (hEPO), Influenza A virus HA (A/Puerto Rico/8/1934 (H1N1)), firefly luciferase (Fluc), and the SARS‐CoV‐2 Omicron variant spike protein, were cloned into the multicloning site. In vitro transcription of mRNA was carried out using the EZ T7 High Yield In Vitro Transcription Kit (Enzynomics, Daejeon, Republic of Korea). mRNA capping was performed using the SC101 system (ST Pharm Co., Ltd., Seoul, Republic of Korea). N1‐methylpseudouridine (TriLink BioTechnologies, San Diego, CA, USA) was substituted for uridine‐5′‐triphosphate (UTP) to enhance stability and reduce immunogenicity. The transcription reaction was incubated overnight at 37°C, followed by DNase I treatment to remove template DNA. mRNA transcripts were precipitated using lithium chloride, washed with 70% ethanol, and resuspended in RNase‐free water. Further purification steps were implemented to reduce double‐stranded RNA contaminants. RNA concentration was determined using a NanoDrop 2000 spectrophotometer (Thermo Fisher Scientific, Waltham, MA, USA). The resulting mRNAs were named CUK3‐1 hEPO, CUK3‐1 HA, CUK3‐1 Fluc, and CUK3‐1 S Omicron, corresponding to the proteins they encoded.

### 4.4 Formulation of mRNA‐Loaded LNPs

TM disulfide‐containing ionizable lipids were synthesized internally at KL's Organic Synthesis Laboratory. DSPC, DOPE, cholesterol, and DMG‐PEG 2K were sourced from Avanti Polar Lipids (Alabaster, AL, USA), whereas SM‐102 was obtained from Hanmi Fine Chemical Co., Ltd. (Siheung, Gyeonggi‐do, Republic of Korea). Lipid components, namely, helper phospholipids, sterols, and PEGylated lipids, were dissolved in a 1:1 (v/v) chloroform/methanol mixture at a 50 µg/µL concentration. These mixtures were combined in predetermined molar ratios and dried under reduced pressure to produce a thin lipid film. The lipid film was subsequently rehydrated in ethanol. mRNA was dissolved in a 50 mM citrate buffer (pH 4.0) at a volume ratio of 1:3 (mRNA:lipid solution). LNPs encapsulating mRNA were prepared via microfluidic mixing using the enCell system (enParticle, Busan, Republic of Korea). The formulations were dialyzed against 1× DPBS to remove ethanol and concentrated using Amicon Ultra‐15 centrifugal filter units (Merck Millipore, Darmstadt, Germany).

### 4.5 Physicochemical Characterization of mRNA‐LNPs

Particle size (Z‐average), PDI, and zeta potential were measured using a Zetasizer Nano ZS (Malvern Instruments, Malvern, UK). Samples were diluted 1:100 in 1× DPBS for size and PDI assessments, and in deionized water for zeta potential measurements. Measurements were performed using 12 mm square polystyrene cuvettes (DTS0012) for size and PDI, and folded capillary zeta cells (DTS1070) for zeta potential.

The structures of the LNPs were observed using Cryo‐TEM. Cryogenic samples were prepared by loading LNP solution onto a glow‐discharged lacey carbon grid (Lacey Carbon, 200 mesh Cu, Ted Pella Inc., USA) that was treated at 15 mA for 60 s to enhance hydrophilicity. The grid was loaded into the Vitrobot Mark IV (Thermo Fisher Scientific), and the chamber of the Vitrobot was maintained at 15°C and 100% humidity. A 3 µL aliquot of the LNP solution was mounted on the grid, and excess liquid was removed by blotting. The blotted specimen was plunge frozen in liquid ethane and stored in liquid nitrogen before cryo‐TEM imaging. The cryogenic sample was transferred to a transmission electron microscope (TEM) (200 kV Cryo‐TEM, Thermo Fisher Scientific, USA, Cooperative Center for Research Facilities, CUK Sungsim Industry‐Academic Cooperation, NFEC‐2026‐03‐315004). Images were acquired at an acceleration voltage of 200 kV with a total electron dose of ∼15 e^−^/Å^2^. For stability assessment, S Omicron mRNA‐ LNPs were incubated under storage and stress conditions, including 4°C, room temperature, and 37°C. Particle size, PDI, and EE were measured on days 1, 3, 4, 7, and 14.

### 4.6 Encapsulation Efficiency Measurement

EE was determined using the RiboGreen RNA assay (Invitrogen, Carlsbad, CA, USA). LNP samples were diluted and either lysed with 0.5% Triton X‐100 in Tris‐EDTA buffer or left unlysed. Following a 200‐fold dilution in Tris‐EDTA buffer, samples were incubated with RiboGreen dye, and fluorescence was measured (excitation 485 nm, emission 520 nm) using a Synergy H1 microplate reader (BioTek, Winooski, VT, USA). EE was calculated relative to a standard curve constructed using free mRNA.

### 4.7 6‐(*p*‐Toluidino)‐2‐naphthalenesulfonic Acid(TNS) Assay

The apparent pKa of LNP was measured using the TNS [6‐(*p*‐toluidino)‐2‐naphthalenesulfonic acid sodium salt] (Sigma‐Aldrich, St. Louis, MO, USA) assay, in accordance with previously reported methods [38, 67]. A buffer solution consisting of 150 mM sodium chloride, 20 mM sodium phosphate, 20 mM ammonium acetate, and 25 mM ammonium citrate (Sigma‐Aldrich, St. Louis, MO, USA) was prepared, and then adjusted in 0.5 pH increments from pH 2 to pH 12 to produce a total of 21 different pH solutions. Each formulated LNP was diluted to a total lipid concentration of 500 µM before use. To a black 96‐well plate, 90 µL of each pH solution and 5 µL of the LNP formulation were added and mixed; all conditions were performed in duplicate. Subsequently, 5 µL of TNS was added to each well to achieve a final TNS concentration of 4.15 µM. The plate was then incubated at room temperature for 20 min, after which fluorescence intensity was measured using a plate reader (SYNERGY MX, BioTek, Vermont, USA) (excitation, 320 nm; emission, 450 nm). For each sample, the average fluorescence value of the pH 12 well was subtracted from the fluorescence values of all pH conditions to calculate the average relative fluorescence at each pH. The average relative fluorescence was plotted against pH, and the apparent pKa was calculated as the pH corresponding to 50% of the maximum relative fluorescence value.

### 4.8 Confocal Microscopy Analysis of Intracellular LNP Trafficking

C2C12 myoblast cells were seeded onto eight‐chamber slides at a density of 1 × 10^7^ cells per chamber and incubated for 24 h prior to treatment. LNP formulations were fluorescently labeled with DiD (Invitrogen, USA) and added to the cells for time‐dependent uptake analysis. At the indicated time points, cells were washed with PBS to remove unbound LNPs and stained with Hoechst (Invitrogen, USA) to visualize nuclei. The intracellular distribution of DiD‐labeled LNPs was then analyzed by confocal laser scanning microscopy. DiD and Hoechst signals were detected in the red and blue channels, respectively, and intracellular puncta formation was compared among the LNP formulations over time.

### 4.9 Animals and Immunization

Female ICR mice (6 weeks old), designated for expression and toxicity studies, and BALB/c mice intended for immunogenicity assessments were sourced from Dae‐Han Biolink (Eumseong, Chungcheongbuk‐do, Republic of Korea). Simultaneously, female BALB/c mice (6 weeks old) for luciferase biodistribution experiments, along with female C57BL/6 mice (6 weeks old) for immunogenicity evaluations, were obtained from Koatech (Gyeonggi‐do, Republic of Korea). All animals were acclimated for one week under specific pathogen‐free conditions and maintained at 23 ± 2°C under a 12/12‐h light/dark cycle. All procedures conformed to protocols approved by the Institutional Animal Care and Use Committee of the Catholic University of Korea (Approval Nos. CUK‐IACUC‐2024‐033 and CUK‐IACUC‐2025‐021).

For immunogenicity studies, BALB/c mice were randomly allocated into groups (*n* = 5 per group) and intramuscularly injected with either 10 µg HA mRNA or 20 µg S Omicron mRNA, each encapsulated within LNPs. Immunization followed a prime‐boost regimen with a 2‐week interval. Blood and spleen samples were harvested post‐euthanasia for downstream immunological analyses. C57BL/6 mice were assigned to groups (*n* = 5 per group) and administered 20 µg of S Omicron mRNA LNPs via intramuscular injection. Animals were euthanized 24 h post‐administration, and spleen and injection‐site tissues were collected for subsequent analyses.

### 4.10 Serum hEPO Expression and Cytokine Analysis

ICR mice received intramuscular injections of CUK3‐1 hEPO mRNA‐loaded LNPs (10 µg in 40 µL). After 6 h, the animals were euthanized, and serum was collected. hEPO levels were quantified using the hEPO DuoSet ELISA Kit (R&D Systems, Minneapolis, MN, USA). Pro‐inflammatory cytokines MCP‐1 and IL‐6 were measured using uncoated ELISA kits (Invitrogen and Thermo Fisher Scientific, Waltham, MA, USA).

### 4.11 ELISA

Ninety six‐well plates were coated with 100 ng/well of antigenic protein corresponding to the mRNA‐encoded antigen (Influenza A H1N1 HA from Sino Biological, Beijing, China, or SARS‐CoV‐2 BA.4/BA.5 Spike RBD protein from Sino Biological). Plates were washed with phosphate‐buffered saline (PBS) containing 0.05% Tween‐20, blocked with 1% bovine serum albumin in PBS, and incubated with serially diluted serum samples starting at a 1:100 dilution. Bound antibodies were detected using horseradish peroxidase‐conjugated anti‐mouse IgG1 and IgG2c secondary antibodies (Invitrogen and Novus Biologicals, Centennial, CO, USA). Tetramethylbenzidine substrate was used for color development, and absorbance was measured at 450 nm using a GloMax Explorer microplate reader (Promega, Madison, WI, USA). Endpoint titers were calculated based on a 95% confidence interval cut‐off [68].

Splenocytes isolated from immunized mice were cultured (5 × 10^5^ cells/well) and stimulated with 500 ng/well HA peptide mix for 72 h at 37°C. Secreted IFN‐γ, TNF‐α, and IL‐2 were measured using ELISA kits (Invitrogen and Thermo Fisher Scientific) according to the manufacturer's instructions.

### 4.12 ELISpot Assay

Splenocytes (5 × 10^5^ cells/well) were stimulated with HA peptides or S Omicron peptides (synthesized by Peptron, Daejeon, Republic of Korea) for 48 h. IFN‐γ‐secreting T cells were detected using the Mouse IFN‐γ ELISpot BASIC kit (Mabtech, Stockholm, Sweden) according to the manufacturer's protocol.

### 4.13 Neutralization Assay

For HA mRNA immunization, neutralizing antibody titers were measured using an HI assay based on established protocols [69]. Mouse sera were treated with receptor‐destroying enzyme (Denka Seiken, Tokyo, Japan), heat‐inactivated, and adsorbed with chicken red blood cells to reduce nonspecific activity. Serial dilutions were incubated with standardized influenza virus, followed by the addition of chicken RBCs. Titers were reported as the reciprocal of the highest dilution inhibiting hemagglutination, with a lower detection limit of 1:10.

For S Omicron mRNA immunization, serum neutralization (SN) assays were performed using SARS‐CoV‐2 virus (10^3^ TCID_50_/mL) following established protocols [70, 71, 72]. Serial two‐fold serum dilutions starting at 1:32 were tested in duplicate.

### 4.14 Toxicological Assessment in Mice

All animal experimental procedures were approved by the Institutional Animal Care and Use Committee of Seoul National University Hospital (Approval Nos. 22‐0088 and 25‐0076). Six‐week‐old CrlOri:CD1 (ICR) mice (Orient Bio Inc., Seongnam, Gyeonggi‐do, Republic of Korea) were housed in individually ventilated cages under controlled conditions (21–25°C; 30%–70% relative humidity). Animals were maintained in a specific‐pathogen‐free facility and acclimated for one week before experimentation. The mice were intramuscularly vaccinated in the left quadriceps muscle with S Omicron mRNA LNP formulations (50 µg/100 µL/mouse) or an equal volume of DPBS (WELGENE, Gyeongsan‐si, Gyeongsangbuk‐do, Republic of Korea) as a control. The vaccination regimen consisted of a primary dose followed by a booster dose at a 2‐week interval. Euthanasia was performed 2 days after the booster dose.

### 4.15 Safety Assessment

*Animal Experiment*: All experimental procedures involving cynomolgus macaques were reviewed and approved by the Institutional Animal Care and Use Committee of Seoul National University Hospital (Approval No. 23‐0366).

Female cynomolgus macaques (*Macaca fascicularis*), aged 4–5 years, were obtained from Orient GENIA Inc. (Seongnam, Kyonggi‐do, South Korea) and housed at the Nonhuman Primate Research Center of Seoul National University Hospital under standardized conditions (temperature 20–28°C; humidity 30%–70%; 12‐h light/dark cycle). The monkeys were provided with Teklad Global 20% Protein Primate Diet (Inotiv, West Lafayette, IN, USA) supplemented with fresh fruit. Animals were intramuscularly vaccinated in the left brachialis muscle with the S Omicron mRNA LNP formulations (800 µg/800 µL/monkey) or an equal volume of DPBS as a control. The vaccination regimen consisted of a primary dose followed by three booster doses at a 2‐week interval. Euthanasia was performed 2 days after the final booster dose.

*Complete Blood Count*: Blood was obtained from the caudal vena cava under isoflurane anesthesia and promptly transferred into K_2_ EDTA‐coated collection tubes (BD Biosciences, Franklin Lakes, NJ, USA). CBC parameters, including RBCs count, hemoglobin, hematocrit, reticulocyte count, lymphocyte count, and platelet count, were obtained using an ADVIA 2120i automated hematology analyzer (Siemens Diagnostics, Tarrytown, NY, USA).

*Serum Biochemistry*: Blood was collected into serum separating tubes (BD Biosciences) and centrifuged at 2000× *g* for 10 min. Serum biochemical analyses for CRP, LDH, HDL‐C, BUN, glucose, AST, and ALT were performed using a 7070 automated chemistry analyzer (Hitachi, Tokyo, Japan).

*Histopathological Analysis*: After necropsy, the collected tissues were fixed in 10% neutral‐buffered formalin. Sternum samples were decalcified for 24 h using Calci‐Clear Rapid solution (National Diagnostics, Charlotte, NC, USA), and then processed through standard histological procedures, embedded in paraffin, sectioned at a thickness of 4 µm, and stained with hematoxylin and eosin. The histopathological evaluation was performed by a trained veterinary pathologist from the laboratory animal pathology. The myeloid‐to‐erythroid (M/E) cell ratio was determined using three representative high‐power fields selected from hematopoietically active bone marrow regions while avoiding fat‐rich or osseous areas. A minimum of 500 myeloid lineage cells (myeloblasts, promyelocytes, myelocytes, metamyelocytes, band forms, and segmented neutrophils) and erythroid precursors were manually counted. The M/E ratio was determined by dividing the total count of myeloid lineage cells by that of erythroid precursors. Injection‐site inflammation in the brachialis muscle was graded on a severity scale from 0 to 5, based on the extent of inflammatory cell infiltration and the proportion of tissue affected.

### 4.16 Biodistribution Analysis

*mRNA Quantification Using RT‐qPCR*: Female cynomolgus monkeys (*n* = 3 per group) were used to evaluate vaccine mRNA biodistribution. Animals received four intramuscular administrations of the mRNA vaccine candidate at 2‐week intervals. Forty‐eight hours after the fourth dose, 14 tissues were collected for RNA extraction: injection site, brain, liver, spleen, kidney, ovary, lung, heart, whole blood, adrenal gland, axillary lymph node (left/right), and distal axillary lymph node (left/right). Total RNA was isolated from each tissue using TRIzol Reagent (Invitrogen) according to the manufacturer's instructions. Vaccine‐derived mRNA was quantified using the StepOnePlus Real‐Time PCR System (Thermo Fisher Scientific). Reactions were prepared using TaqMan Universal Master Mix II, with UNG, and run under standard cycling conditions recommended for TaqMan assays using the following primers: forward primer (5′‐GAGGCGATGAGGTGAGACAGAT‐3′); reverse primer (5′‐GAAGTCGTCGGGCAGCTTAT‐3′); TaqMan Probe (FAM‐ACAGGCAATATCGCC‐BHQ1). The mRNA copy number was calculated using the following formula:

$$Copy\;number=\frac{X\;\left(ng\right)\times 6.0221\times 10^{23}\left(\frac{molecules}{mol}\right)}{N\times 321.9\left(\frac{g}{mol}\right)\times 10^{9}\left(\frac{ng}{g}\right)}$$

where *X* = amount of RNA (ng), *N* = RNA length (nt), 6.0221 × 10^2^ ^3^ = Avogadro's number, and 321.9 g/mol = average molecular weight per 1 bp RNA.

### 4.17 FLuc Distrubustion for In Vivo Expression

BALB/c mice were immunized intramuscularly with 10 µg in 40 µL Fluc mRNA‐LNP. After 3 h, the mice were injected intraperitoneally (IP) with 3 mg in 100 µL of luciferin in saline (Promega Inotiv) and sacrificed 5 min later. The spleen, liver, lymph node, kidney, heart, lung, and injection site were harvested, and Fluc biodistribution in these organs was assessed using the IVIS Spectrum System (LUCI, CELLGENTEK). Image analysis was performed using Living Image software (NEOimage program).

### 4.18 RT‐qPCR

*RNA Extraction*: Organ samples collected in each experiment were stored at −80°C. Subsequently, the samples were chopped, placed in TRIzol (Thermo Fisher Scientific), and homogenized on ice. Chloroform (200 µL) was added, and the mixture was vortexed for 10 s and incubated at 25°C for 10 min. The mixture was then centrifuged at 13500 rpm for 15 min at 4°C, and 600 µL of the aqueous phase was transferred to a new tube. Subsequently, 600 µL of isopropanol and 1 µL of glycoblue (Invitrogen) were added, the mixture was gently inverted, and then centrifuged at 13500 rpm for 15 min at 4°C. The supernatant was removed, and the RNA pellet was washed with 70% ethanol dissolved in water. The mixture was centrifuged at 13500 rpm for 15 min at 4°C, and the supernatant was discarded. The RNA pellet was air‐dried for 15 min, dissolved in RNase‐free water, and stored at −80°C until cDNA synthesis.

*cDNA Synthesis*: cDNA was synthesized using the ReverTra Ace qPCR RT Kit & Master Mix (TOYOBO, Osaka, Japan), with 800 ng of total RNA used to generate 40 µL of cDNA. RNA concentration was measured spectrophotometrically using a NanoDrop 2000 spectrophotometer (Thermo Fisher Scientific).

*PCR*: PCR was performed using TB Green Premix Ex Taq II–Tli RNaseH Plus (TAKARA) according to the manufacturer's instructions. Data were analyzed using the 2^−ΔΔCT^ method. Primers for quantitative reverse transcription PCR are presented in Table S1.

### 4.19 Transmission Electron Microscopy for Structural Analysis of Mitochondria

TEM imaging was performed to analyze mitochondrial structure in muscle tissues. Muscle tissues treated with DPBS (NC), ALC‐0315‐based LNP (ALC), or TM1‐OPT3‐C were harvested and sectioned into 1 mm^3^ sections. The sectioned muscle tissues were fixed in 4% paraformaldehyde solution for 24 h at 4°C, followed by rinsing three times with 0.05 M sodium cacodylate buffer. The tissues were post‐fixed with 1% osmium tetroxide in 0.1 M sodium cacodylate buffer for 1 h at room temperature and washed three times with deionized water. Following post‐fixation, the samples were dehydrated through a graded ethanol (30, 50, 70, 80, 90, and three changes of 100% in sequence, 15 min each), followed by incubation in propylene oxide solution three times for 15 min each. The dehydrated samples were immersed in graded Spurr's resin (EMS, USA) mixed with propylene oxide solution at ratios of 1:1, 2:1, and 100% for 1.5 h each. The samples were embedded in freshly prepared resin and cured overnight at 70°C. The tissue‐embedded resin was sectioned at approximately 100 nm thickness using an ultramicrotome (EM UC7, Leica, Germany). The sections were mounted on a carbon‐film TEM grids (TED PELLA. Inc., USA), and the mitochondria within the muscle tissues were observed using a TEM (JEM‐2100F, JEOL, Japan) at an accelerating voltage of 200 kV.

### 4.20 Statistical Analysis

Data analysis was performed using GraphPad Prism 8 (GraphPad Software, San Diego, CA, USA). Data are expressed as the mean ± standard deviation (SD). Comparisons between two groups were conducted using unpaired Student's *t*‐tests, whereas comparisons among multiple group were conducted using one‐way or two‐way ANOVA with Tukey–Kramer post hoc tests. Significance thresholds were set at *p < 0.05, **p < 0.01, ***p < 0.001, and ****p < 0.0001.

## Author Contributions

**S.H.B**, **J.L**., **J.H.A**, **H.C**., **H.C**., **J.K**., **M.K**.: writing – review and editing, writing – original draft, visualization, investigation, formal analysis, data curation, conceptualization, methodology, software, validation. **H.R.O**., **S.H.P**., **E.Y.O**.: visualization, validation, software, methodology, investigation, data curation. **S.J**., **Y.L**., **Y.S.L**., **N.Y.L**., **H.J.B**., **J.K**., **J.K**., **N.L**., **B.K**., **J.H.K**., **E.S**., **D.S**., **H.Y**.: methodology, investigation. **J.L**., **S.M.L**., **H.Y**., **K.L**., **B.C.K**., **J.H.N**.: writing – review and editing, writing – original draft, visualization, supervision, resources, project administration, methodology, investigation, funding acquisition, conceptualization.

## Conflicts of Interest

The authors declare no conflict of interest.

## Supporting information




**Supporting File**: advs76539‐sup‐0001‐SuppMat.docx.

## Data Availability

The data that support the findings of this study are available from the corresponding author upon reasonable request.
