# Supplementary material for: Optimized Lipid Nanoparticles with Tail‐Modified Ionizable Lipids for Safer mRNA Delivery
Source: Adv Sci (Weinh). 2026 Jul 14;13(55):e76539. doi: 10.1002/advs.76539 (PMC13367110; doi:10.1002/advs.76539)
Supplement: Supplementary file 1 [file ADVS-13-e76539-s001.docx]

Supporting Information

**Optimized Lipid Nanoparticles with Tail-Modified Ionizable Lipids for Safer mRNA Delivery**

Seo-Hyeon Bae^1,2,^**^†^**, Jisun Lee^1,^**^†^**, Jae-Hun Ahn^3,^**^†^**, Hosam Choi^4,^**^†^**, Huijeong Choi^1,2,^**^†^**, Jungmin Kim^1,2,^**^†^**, Minho Kang^2,5,6,^**^†^**, Ho Rim Oh^7,8^, So Hyun Park^9^, Eun Young Oh^9^, Sanghyuk Jeon^1,2^, Yeeun Lee^1,2^, Yu-Sun Lee^1,2^, Na-Young Lee^3,10^, Hee-Jin Bae^3,11^, Jina Kwak^3^, Jooil Kim^3^, Nakyung Lee^4^, Beomsun Kang^4^, Ji-Hye Kim^2,5,6^, Eunsaem Song^2,5,6^, Daesub Song^12^, Hyunho Yoon^1,2^, Sang-Myeong Lee^9,^**^*^**, Hyewon Youn^7.8,^**^*^**, Kiyoun Lee^4,^**^*^**, Byeong-Cheol Kang^3,11,^**^*^**, Jae-Hwan Nam^1,2,13,^**^*^**

**Supplementary Figures and Figure legends**

**Figure S1**

**Figure S1. Apparent pKa measurement of representative LNP formulations by TNS assay.** Normalized TNS fluorescence intensity was measured over a pH range of 2.0–12.0. Apparent pKa values were calculated as the pH corresponding to 50% of the maximum fluorescence intensity. The table summarizes the composition and calculated apparent pKa values of the representative LNP formulations.

**Figure S2**

**Figure S2. In vivo analyses of immune responses of ORI and Positive control of the SM-102-based LNP**

Mice were immunized with saline or HA mRNA-LNPs (10 µg/40 µL) as a prime dose, followed by a booster of the same dose 2 weeks later to compare immune responses. Serum samples were collected 2 weeks after the booster. **(a)** Serum IgG1 and IgG2a levels measured using ELISA. **(b)** Hemagglutination inhibition (HI) titers against vaccine strains measured in serum using the HI assay. **(c)** Numbers of HA peptide-specific IFN-γ-secreting cells in splenocytes measured using ELISpot. **(d)** IFN-γ and TNF-α concentrations measured in splenocyte culture supernatants using ELISA. Data are reported as the mean ± standard deviation (SD). Statistical significance was analyzed using one-way ANOVA. Differences were considered significant at *P < 0.05, **P < 0.01, ***P < 0.001, and ****P < 0.0001. Asterisks without connecting lines indicate comparisons with the N.C. group. All graphical illustrations were created using BioRender.com. (SM: SM-102-based LNP, ORI: Original LNP)

**Figure S3**

**Figure S3. Physicochemical characterization of mRNA-LNP formulations.** **(a)** Particle size and polydispersity index (PDI) measurements of selected LNP candidates. **(b)** Surface charge (zeta potential) and mRNA encapsulation efficiency (EE) of the LNPs. Data were analyzed using one-way ANOVA, with significance levels indicated as *p < 0.05, **p < 0.01, and ***p < 0.001, Compared with the P.C. group. (SM: SM-102-based LNP, ORI: original LNP, EE: Encapsulation Efficiency)

**Figure S4**

**Figure S4.** **Preliminary in vivo screening of mRNA expression and cytokine induction using rationally designed LNPs with varying ionizable lipid compositions.**

**(a)** Schematic illustration of the experimental workflow used for the rational optimization of LNP formulations containing 25% ionizable lipid, including hEPO mRNA encapsulation and single-dose immunization. Seven-week-old ICR mice were intramuscularly injected with hEPO mRNA-LNPs (10 µg/40 µL), and serum samples were collected up to 6 h post-immunization for ELISA analysis. **(b)** Relative serum levels of hEPO protein expressed from mRNA-LNPs with different lipid compositions. **(c)** Relative serum concentrations of MCP-1 and IL-6 following administration of hEPO mRNA-LNPs.

Data are presented as mean ± SD and normalized to the corresponding LNP ORI group included in each experiment. Statistical significance was determined by one-way ANOVA. (*P < 0.05, **P < 0.01, ***P < 0.001, ****P < 0.0001, compared with the LNP ORI group) (ORI: Original LNP)

**Figure S5**

**Figure S5.** Evaluation of mRNA-loaded LNP candidates. **(a)** Size and polydispersity index (PDI) of the eight LNP formulations tested. **(b)** Zeta potential and encapsulation efficiency (EE) of the mRNA within the LNPs. Statistical significance was determined by one-way ANOVA and defined as *p < 0.05, **p < 0.01, and ***p < 0.001, compared with the SM group. (SM: SM-102-based LNP, ORI: Original LNP, EE: Encapsulation Efficiency)

**Figure S6**

**Figure S6.** Serum neutralization (SN) titer against vaccine strains measured using neutralization assay with pooled serum samples collected 2 weeks after the booster. (ORI: Original LNP)

**Figure S7**

**Figure S7. Time-dependent intracellular trafficking of DiD-labeled LNP formulations in C2C12 cells.** C2C12 cells were treated with DiD-labeled LNP formulations (ORI, TM1, and TM1-OPT3-C), and intracellular localization was visualized by confocal microscopy at the indicated time points (6, 9, 12, 16, and 24 h). Cell nuclei were stained with Hoechst (blue), and DiD-labeled LNPs are shown in red. White arrows indicate representative intracellular puncta structures. ORI and TM1 exhibited prominent puncta formation primarily between 6–12 h post-treatment, whereas TM1-OPT3-C showed delayed but sustained puncta accumulation, which was most evident between 12–16 h. These punctate structures are consistent with intracellular vesicular localization following endocytic uptake and suggest formulation-dependent differences in intracellular trafficking kinetics. Scale bars = 10 μm. (ORI: Original LNP)

**Figure S8**

**Figure S8. Stability evaluation of representative mRNA-LNP formulations under different storage conditions.**

The physicochemical stability of SM, ORI, TM1, and TM1-OPT3-C formulations was monitored for 14 days at 4 °C, room temperature (RT), and 37 °C. Changes in particle size (top row), polydispersity index (PDI; middle row), and encapsulation efficiency (EE; bottom row) were evaluated at the indicated time points. All formulations maintained relatively stable particle sizes, low PDI values, and high encapsulation efficiencies throughout the study period under all tested conditions, indicating favorable colloidal stability and mRNA encapsulation stability. Data are presented as mean ± SD (n = 3).

**Figure S9**

**Figure S9.** **Stability characterization of the mRNA-loaded LNP formulations under different storage periods.** Cryo-TEM images of mRNA-loaded LNP stored at 4 ℃ for 1, 7, and 14 days in a pH 7.4 buffer.

**Figure S10**

**Figure S10.** **pH-dependent structural transitions of the mRNA-loaded LNP formulations under different pH conditions.** Cryo-TEM images of mRNA-loaded LNP incubated at 4 °C under pH 7.4, 6.0, and 5.0.

Supplementary Methods

1. Synthesis of Ionizable Lipids

**1.1. General Methods.**

All reactions were conducted in oven-dried glassware under nitrogen. Unless otherwise stated, all reagents were purchased from Sigma–Aldrich, Acros, or Fisher and were used without further purification. All solvents were ACS grade or better and used without further purification. Analytical thin layer chromatography (TLC) was performed with glass backed silica gel (60 Å) plates with fluorescent indication (Whatman). Visualization was accomplished by UV irradiation at 254 nm and/or by staining with ninhydrin, phosphomolybdic acid (PMA) solution or *p*-anisaldehyde solution. Flash column chromatography was performed by using silica gel (particle size 70–230 mesh ASTM). All ^1^H NMR and ^13^C NMR spectra were recorded at 298 K on a Bruker Avance III HD 500 (500 MHz) spectrometer in CDCl_3_ by using the signal of residual CHCl_3_ as an internal standard. All NMR *δ* values are given in ppm, and all *J* values are in Hz.

**2. Experimental Procedure**

**2.1. Synthesis of disulfide bond tail (C_2_(S-S)C_14_) precursor (1)**

**Compound 1.** A solution of 2-mercaptoethanol (3.0 mL, 32.7 mmol) and 1-tetradecanethiol (8.7 mL, 32.7 mmol, 1 equiv) in CH_2_Cl_2_:MeOH = 3:1 (total: 100 mL, 0.3 M) was treated with I_2_ (8.3 g, 32.7 mmol, 1 equiv) and pyridine (5.3 mL, 65.4 mmol, 2 equiv). After stirring for 12 h at 25 °C, the resulting mixture was concentrated in vacuo. The white solid was diluted with H_2_O (100 mL) and CH_2_Cl_2_ (100 mL). The layers were separated, and the aqueous layer was extracted with CH_2_Cl_2_ (100 mL×2). The combined organic layers were washed with saturated aqueous NaCl (100 mL×2), dried over anhydrous Na_2_SO_4_, and concentrated in vacuo. The residue was purified by column chromatography (SiO_2_, gradient eluent, 5–20% EtOAc/hexane) to provide **1** (5.1 g, 51%) as a white solid.

**2.2. Synthesis of EB-5T-1 Chain**

**EB-5T-1.** A solution of glutaric anhydride (1.88 g, 46.4 mmol, 1.5 equiv) and 2-hexyl-1-decanol (7.5 g, 30.9 mmol) in CH_2_Cl_2_ (52 mL, 0.6 M) was treated with DMAP (9.4 g, 77 mmol, 2.5 equiv). After strring for 9 h, the resulting mixture was diluted with H_2_O (50 mL) and acidified with 6M HCl at 0 °C. The layers were separated, and the aqueous layer was extracted with CH_2_Cl_2_ (50 mL×2). The combined organic layers were washed with saturated aqueous NaCl (100 mL×1), dried over anhydrous Na_2_SO_4_, and concentrated in vacuo. The residue was purified by column chromatography (SiO_2_, gradient eluent, 5–15% EtOAc/hexane) to provide **EB-5T-1** (10 g, 91%) as a colorless oil.

**2.3. Representative synthetic method for *ionizable lipid* TM 1**

**Acetonide protection**: synthesis of (*R*)-3-(2,2,5,5-tetramethyl-1,3-dioxane-4-carboxamido)propanoic acid **(2)**

A suspension of D-pantothenic acid hemicalcium salt (5.0 g, 21 mmol) in DMF (42 mL, 0.5 M) was treated with (MeO)_2_C(CH_3_)_3_ (210 mL, 0.1 M) and *p*-TsOH·H_2_O (4.4 g, 23 mmol, 1.1 equiv). After stirring for 7 h at 25 °C, the resulting mixture was filtered through a pad of celite. Add toluene to the filterate to form an azeotrope with DMF and the resulting mixture was concentrated in vacuo. The resulting a pale yellow solid was directly used for next step with no further purification.

**EDC/DMAP coupling reaction**: synthesis of 2-(tetradecyldisulfaneyl)ethyl (*R*)-3-(2,2,5,5-tetramethyl-1,3-dioxane-4-carboxamido)propanoate **(3)**

A cooled (0 °C) solution of acid **2** (2.9 g, 11.0 mmol, 1.3 equiv) and EDC hydrochloride (2.4 g, 13 mmol, 1.5 equiv) and DMAP (207 mg, 1.70 mmol, 20 mol%) in CH_2_Cl_2_ (21 mL, 0.4 M) was stirred for 30 min before alcohol **1** (2.6 g, 8.5 mmol) in CH_2_Cl_2_ (10 mL) was added to the above solution. After stirring for 7 h at 25 °C, the resulting mixture was diluted with saturated aqueous NaHCO_3_ (20 mL) and CH_2_Cl_2_ (20 mL). The layers were separated, and the aqueous layer was extracted with CH_2_Cl_2_ (20 mL×2). The combined organic layers were washed with saturated aqueous NaCl (30 mL×1), dried over anhydrous Na_2_SO_4_, and concentrated in vacuo. The residue was purified by column chromatography (SiO_2_, gradient eluent, 10–33% EtOAc/hexane) to provide **3** (4.36 g, 94%) as a colorless oil.

**Acetonide deprotection**: synthesis of 2-(tetradecyldisulfaneyl)ethyl (*R*)-3-(2,4-dihydroxy-3,3-dimethylbutanamido)propanoate **(4)**

A cooled (0 °C) solution of **3** (2.5 g, 4.6 mmol) in MeCN:H_2_O = 1:1 (total: 10 mL, 0.46 M) was treated with 2,2,2-trifluoroacetic acid (TFA, 5.0 mL, 0.92 M). After stirring for 30 min at 25 °C, the resulting mixture was diluted with H_2_O (20 mL) and CH_2_Cl_2_ (20 mL) before it was basified with saturated aqueous NaHCO_3_ at 25 °C. The layers were separated, and the aqueous layer was extracted with CH_2_Cl_2_ (50 mL×3). The combined organic layers were washed with saturated aqueous NaCl (50 mL×1), dried over anhydrous Na_2_SO_4_, and concentrated in vacuo. The residue was purified by column chromatography (SiO_2_, gradient eluent, 33–90% EtOAc/hexane) to provide diol **4** (1.88 g, 81%) as a colorless oil.

**EDC/DMAP coupling reaction**: synthesis of (*R*)-3-hydroxy-2,2-dimethyl-4-oxo-4-((3-oxo-3-(2-(tetradecyldisulfaneyl)ethoxy)propyl)amino)butyl 3-(dimethylamino)propanoate **(5)**

A cooled (0 °C) solution of 3-(dimethylamino)propionic Acid Hydrochloride (943 mg, 6.14 mmol, 1.2 equiv) and EDC hydrochloride (1.5 g, 7.7 mmol, 1.5 equiv) and DMAP (125 mg, 1.02 mmol, 20 mol%) in CH_2_Cl_2_ (13 mL, 0.4 M) was stirred for 30 min before diol **4** (2.6 g, 5.1 mmol) in CH_2_Cl_2_ (10 mL) was added to the above solution. After stirring for 7 h at 25 °C, the resulting mixture was diluted with saturated aqueous NaHCO_3_ (20 mL) and CH_2_Cl_2_ (20 mL). The layers were separated, and the aqueous layer was extracted with CH_2_Cl_2_ (20 mL×2). The combined organic layers were washed with saturated aqueous NaCl (30 mL×1), dried over anhydrous Na_2_SO_4_, and concentrated in vacuo. The residue was purified by column chromatography (2% Et_3_N, SiO_2_, gradient eluent, 1–5% MeOH/CH_2_Cl_2_) to provide **5** (2.6 g, 84%) as a colorless oil.

**EDC/DMAP coupling reaction**: synthesis of ***ionizable lipid* TM 1**

A cooled (0 °C) solution of **EB-5T-1** (1.9 g, 5.6 mmol, 1.3 equiv) and EDC hydrochloride (1.2 g, 6.4 mmol, 1.5 equiv) and DMAP (105 mg, 0.856 mmol, 20 mol%) in CH_2_Cl_2_ (11 mL, 0.4 M) was stirred for 30 min before **5** (2.6 g, 4.3 mmol) in CH_2_Cl_2_ (10 mL) was added to the above solution. After stirring for 7 h at 25 °C, the resulting mixture was diluted with saturated aqueous NaHCO_3_ (10 mL) and CH_2_Cl_2_ (10 mL). The layers were separated, and the aqueous layer was extracted with CH_2_Cl_2_ (20 mL×2). The combined organic layers were washed with saturated aqueous NaCl (30 mL×1), dried over anhydrous Na_2_SO_4_, and concentrated in vacuo. The residue was purified by column chromatography (2% Et_3_N, SiO_2_, gradient eluent, 15–60% EtOAc/hexane) to provide ***ionizable lipid* TM 1** (3.07 g, 76%) as a colorless oil; ^1^H NMR (500 MHz, CDCl_3_) δ 7.54 (t, *J* = 5.9 Hz, 1H), 4.80 (s, 1H), 4.32 (t, *J* = 6.7 Hz, 2H), 3.98 (d, *J* = 11.2 Hz, 1H), 3.95 (d, *J* = 5.8 Hz, 2H), 3.74 (d, *J* = 11.2 Hz, 1H), 3.57 (dq, *J* = 12.8, 6.4 Hz, 1H), 3.33 (dq, *J* = 12.4, 6.4 Hz, 1H), 2.86 (t, *J* = 6.7 Hz, 2H), 2.64–2.71 (m, 3H), 2.44–2.57 (m, 7H), 2.36 (t, *J* = 7.4 Hz, 2H), 2.26 (s, 6H), 1.94 (p, *J* = 7.2 Hz, 2H), 1.58–1.67 (m, 3H) , 1.22–1.36 (m, 46H), 1.03 (d, *J* = 11.9 Hz, 6H), 0.85 (t, *J* = 6.6 Hz, 9H); ^13^C NMR (125 MHz, CDCl_3_) δ 173.1, 172.1, 171.9, 171.7, 167.9, 76.6, 69.6, 67.3, 62.7, 55.2, 45.2, 39.1, 37.25, 37.16, 36.9, 35.0, 33.8, 33.2, 33.11, 33.07, 31.91, 31.88, 31.8, 31.2, 29.9, 29.68, 29.66, 29.64, 29.60, 29.59, 29.56, 29.5, 29.349, 29.30, 29.2, 29.1, 28.5, 26.7, 26.6, 22.68, 22.67, 22.6, 22.2, 20.1, 20.0, 14.12, 14.10; HRMS (FAB, *m*/*z*): [M+H]^+^ calcd for C_51_H_97_N_2_O_9_S_2_ 945.6635; found: 945.6631.

**EDC/DMAP coupling reaction**: synthesis of ***ionizable lipid* TM 2**

A cooled (0 °C) solution of **EB-6T-1** (726 mg, 1.96 mmol, 1.5 equiv) and EDC hydrochloride (501 mg, 2.61 mmol, 2 equiv) and DMAP (32 mg, 0.261 mmol, 20 mol%) in CH_2_Cl_2_ (3.3 mL, 0.4 M) was stirred for 30 min before **5** (793 mg, 1.31 mmol) in CH_2_Cl_2_ (2 mL) was added to the above solution. After stirring for 7 h at 25 °C, the resulting mixture was diluted with saturated aqueous NaHCO_3_ (5 mL) and CH_2_Cl_2_ (5 mL). The layers were separated, and the aqueous layer was extracted with CH_2_Cl_2_ (10 mL×2). The combined organic layers were washed with saturated aqueous NaCl (20 mL×1), dried over anhydrous Na_2_SO_4_, and concentrated in vacuo. The residue was purified by column chromatography (2% Et_3_N, SiO_2_, gradient eluent, 15–60% EtOAc/hexane) to provide ***ionizable lipid* TM 2** (902 mg, 72 %) as a colorless oil; ^1^H NMR (500 MHz, CDCl_3_) δ 7.52 (t, *J* = 5.7 Hz, 1H), 4.83 (s, 1H), 4.34 (t, *J* = 6.7 Hz, 2H), 4.00 (d, *J* = 11.2 Hz, 1H), 3.95 (d, *J* = 5.8 Hz, 2H), 3.77 (d, *J* = 11.2 Hz, 1H), 3.59 (dq, *J* = 12.7, 6.3 Hz, 1H), 3.36 (dq, *J* = 12.5, 6.4 Hz, 1H), 2.88 (t, *J* = 6.7 Hz, 2H), 2.67–2.73 (m, 3H), 2.50–2.60 (m, 5H), 2.42-2.45 (m, 2H), 2.28-2.33 (m, 8H), 1.60-1.67 (m, 7H), 1.25-1.37 (m, 46H) , 1.05 (d, *J* = 9.6 Hz, 6H), 1.03 (d, *J* = 11.9 Hz, 6H), 0.87 (t, *J* = 6.6 Hz, 9H); ^13^C NMR (125 MHz, CDCl_3_) δ 173.5, 172.4, 172.0, 171.7, 168.0, 76.5, 69.7, 67.3, 62.7, 55.1, 54.2, 31.93, 31.91, 31.2, 29.70, 29.68, 29.66, 29.62, 29.61, 29.6, 29.4, 22.70, 22.69, 22.66, 14.13, 14.12; HRMS (FAB, *m*/*z*): [M+H]^+^ calcd for C_52_H_99_N_2_O_9_S_2_ 959.6792; found: 959.6782.

**EDC/DMAP coupling reaction**: synthesis of ***ionizable lipid* TM 3**

A cooled (0 °C) solution of **EB-7T-1** (804 mg, 2.09 mmol, 1.5 equiv) and EDC hydrochloride (534 mg, 2.79 mmol, 2 equiv) and DMAP (34 mg, 0.279 mmol, 20 mol%) in CH_2_Cl_2_ (3.5 mL, 0.4 M) was stirred for 30 min before **5** (846 mg, 1.39 mmol) in CH_2_Cl_2_ (2 mL) was added to the above solution. After stirring for 7 h at 25 °C, the resulting mixture was diluted with saturated aqueous NaHCO_3_ (5 mL) and CH_2_Cl_2_ (5 mL). The layers were separated, and the aqueous layer was extracted with CH_2_Cl_2_ (10 mL×2). The combined organic layers were washed with saturated aqueous NaCl (20 mL×1), dried over anhydrous Na_2_SO_4_, and concentrated in vacuo. The residue was purified by column chromatography (2% Et_3_N, SiO_2_, gradient eluent, 15–60% EtOAc/hexane) to provide ***ionizable lipid* TM 3** (1.02 g, 75%) as a colorless oil; ^1^H NMR (500 MHz, CDCl_3_) δ 7.50 (t, *J* = 5.8 Hz, 1H), 4.82 (s, 1H), 4.33 (t, *J* = 6.7 Hz, 2H), 4.00 (d, *J* = 11.2 Hz, 1H), 3.95 (d, *J* = 5.8 Hz, 2H), 3.75 (d, *J* = 11.2 Hz, 1H), 3.59 (dq, *J* = 12.8, 6.3 Hz, 1H), 3.35 (dq, *J* = 12.7, 6.3 Hz, 1H), 2.88 (t, *J* = 6.7 Hz, 2H), 2.66–2.72 (m, 3H), 2.48-2.59 (m, 5H), 2.38-2.43 (m, 2H), 2.28-2.31 (m, 8H), 1.60-1.68 (m, 7H), 1.24-1.39 (m, 48H), 1.05 (d, *J* = 11.1 Hz, 6H), 0.87 (t, *J* = 6.8 Hz, 9H); ^13^C NMR (125 MHz, CDCl_3_) δ 173.5, 172.4, 171.7, 171.4, 167.8, 76.3, 69.4, 66.9, 62.5, 55.0, 45.1, 31.74, 31.71, 31.1, 29.51, 29.49, 29.47, 29.43, 29.41, 22.51, 22.49, 22.5, 13.95, 13.93; HRMS (FAB, *m*/*z*): [M+H]^+^ calcd for C_53_H_101_N_2_O_9_S_2_ 973.6948; found: 973.6952.

**Figure S11.** ^1^H NMR spectrum of ***ionizable lipid* TM 1**

**Figure S12.** ^13^C NMR spectrum of ***ionizable lipid* TM 1**

**Figure S13.** High resolution mass spectrum (HRMS) of ***ionizable lipid* TM 1**

**Figure S14.** ^1^H NMR spectrum of ***ionizable lipid* TM 2**

**Figure S15.** ^13^C NMR spectrum of ***ionizable lipid* TM 2**

**Figure S16.** High resolution mass spectrum (HRMS) of ***ionizable lipid* TM 2**

**Figure S17.** ^1^H NMR spectrum of ***ionizable lipid* TM 3**

**Figure S18.** ^13^C NMR spectrum of ***ionizable lipid* TM 3**

**Figure S19.** High resolution mass spectrum (HRMS) of ***ionizable lipid* TM 3**

**Supplementary Table**

**Table S1. Primers for quantitative reverse transcription PCR**

| **Gene** | **Forward primer** | **Reverse Primer** |
| --- | --- | --- |
| **ND1**^a)^ | 5’- CTAGCAGAAACAAACCGGGC -3’ | 5’- CCGGCTTCGTATTCTACGTT -3’ |
| **HK2**^b)^ | 5’- GCCAGCCTCTCCTGATTTTAGTGT -3’ | 5’-GGGAACACAAAAGACCTCTTCTGG -3’ |
| **PGC-1α** ^c)^ | 5’- CGGAAATCATATCCAACCAG -3’ | 5’- CGGAAATCATATCCAACCAG -3’ |
| **TFAM** ^d)^ | 5’- CTGATGGGTATGGAGAAGGAGG -3’ | 5’- CCAACTTCAGCCATCTGCTCTTC -3’ |
| **MCP-1** ^e)^ | 5’- CAGCCAGATGCAGTTAACG -3’ | 5’- TCTCTCTTGAGCTTGGTGAC -3’ |
| **TNF-α** ^f)^ | 5’- GCCTCTTCTCATTCCTGCTTG -3’ | 5’- CTGATGAGAGGGAGGCCATT -3’ |
| **IL-6** ^g)^ | 5’- ACGGCCTTCCCTACTTCACA -3’ | 5’- CATTTCCACGATTTCCCAGA -3’ |
| **IL-1β** ^h)^ | 5’- GCAACTGTTCCTGAACTCAACT -3’ | 5’- ATCTTTTGGGGTCCGTCAACT -3’ |
| **GAPDH** ^i)^ | 5’- TCCCACTCTTCCACCTTCGA -3’ | 5’- CAGGAAATGAGCTTGACAAAGTTG -3’ |

^a)^ND1, NADH dehydrogenase subunit 1; ^b)^HK2, hexokinase 2; ^c)^PGC-1α, Peroxisome Proliferator-Activated Receptor Gamma Coactivator 1-Alpha; ^d)^TFAM, Mitochondrial transcription factor A; ^e)^MCP-1, Monocyte chemoattractant protein-1; ^f)^TNF-α, Tumor necrosis factor-alpha; ^g)^IL-6, Interleukin-6; ^h)^IL-1β, Interleukin-1 beta; ^i)^GAPDH, glyceraldehyde-3-phosphoate dehydrogenase
